# Distinctive characteristics of prolonged standing low back pain developers’ and the associated risk factors: systematic review and meta-analysis

**DOI:** 10.1038/s41598-023-33590-5

**Published:** 2023-04-19

**Authors:** Fatemeh Khoshroo, Foad Seidi, Mohammad Bayattork, Yousef Moghadas-Tabrizi, Erika Nelson-Wong

**Affiliations:** 1 Health and Sports Medicine Department, Faculty of Sport Sciences and Health, University of Tehran, Tehran, Iran; 2 Sport Sciences and Physical Education, Faculty of Humanities Science, University of Hormozgan, Bandar Abbas, Iran; 3 Department of Physical Therapy, Augustana University, Sioux Falls, SD USA

**Keywords:** Medical research, Risk factors

## Abstract

Pain developers (PDs) are considered a pre-clinical low back pain (LBP) population at risk of clinical LBP development and thus exacting great social and economic costs. Therefore, it is necessary to comprehensively investigate their distinctive characteristics and the risk factors of standing-induced LBP based on which appropriate preventive measures can be planned. Scopus, Web of Science, and PubMed databases as well as Google Scholar and ProQuest were systematically searched from inception through 14 July 2022 using a combination of terms relevant to ‘standing’ and ‘LBP’. Studies with low risk of bias in English and Persian using a methodological quality scoring system were deemed eligible for inclusion if they were laboratory studies using prolonged standing duration greater than 42 min to classify adult PDs and non-pain developers (NPDs) without a history of LBP. PDs were compared with NPDs in demographics, biomechanical, and psychological outcomes. Weighted or standardized mean differences, and Hedge’s g were generated to determine the pooled effect sizes using STATA software version 17. 52 papers and theses involving 1070 participants (528 PDs and 542 NPDs) were eligible for inclusion in the systematic review 33 of which were used in meta-analyses. Significant differences between PDs and NPDs in terms of movement patterns, muscular, postural, psychological, structural, and anthropometric variables were evidenced. The following factors were found to have a statistically significant association with standing-induced LBP: lumbar fidgets (Hedge’s g − 0.72, 95% CI − 1.35 to − 0.08, *P* = 0.03), lumbar lordosis in participants over 25 years (Hedge’s g 2.75, 95% CI 1.89–3.61, *P* < 0.001), AHAbd test (WMD 0.7, 95% CI 0.36–1.05, *P* < 0.001), GMed co-activation (Hedge’s g 4.24, 95% CI 3.18–5.3, *P* < 0.001), and Pain Catastrophizing Scale (WMD 2.85, 95% CI 0.51–5.19, *P* = 0.02). Altered motor control displayed in AHAbd test and higher lumbar lordosis in individuals over 25 years seem to be probable risk factors for standing-induced LBP. In order to detect standing-induced LBP risk factors, future researchers should investigate the association of the reported distinctive characteristics to the standing-induced LBP and that whether they are manipulable through various interventions.

## Introduction

Low back pain (LBP) is a serious health problem exacting great social and economic costs^1^. Approximately, 70–85% of adults will suffer from an acute episode of LBP at some point in their lives^2^. Interestingly, 78% of individuals will have the recurrence of LBP within the first year after the initial onset^3^. Besides, 10–20% of individuals experiencing LBP have shown a progression into chronicity^4,5^. This disorder can lead to disability and functional limitations^6^. In addition, 85% of LBP incidences are classified as ‘‘non-specific’’ and a definitive diagnosis cannot be achieved using current radiological methods^7^ which has further complicated the effective clinical management of this disorder. Given the global growth of individuals suffering from LBP over the coming decades as the population ages^8^, the best approach to managing this problem is to basically prevent it through early identification of individuals prone to LBP.

One proposed approach for early identification of individuals prone to low back pain has been through observing low back pain response to long-duration standing exposures^9–61^. A percentage of asymptomatic participants with no history of LBP will develop LBP enabling the researchers to identify both people prone to LBP and the associated risk factors of standing-induced LBP^41^. Sorensen and colleagues indicated that symptoms experienced during the standing paradigm are similar to symptoms typically experienced by people with LBP^62^ verifying the validity of the paradigm. Also, Nelson-Wong and colleagues showed high repeatability in identifying PDs when tested 4 weeks apart^41^.

Prolonged standing pain developers (PDs) have shown many common characteristics with patients suffering from LBP^15,21,24,29,33,36,37,42,46,50,51,55,62^. In other words, they have been compared either directly to patients with “non-specific”^62^, chronic^36^, and recurrent LBP^16^, or indirectly to non-pain developers (NPDs) based on distinctive characteristics previously reported for patients with LBP in the literature. PDs have displayed altered muscle activation^9,14,24,32,33,39,41,44^, alignment^20,26,35,51,56^, and movement patterns^13,20,29,35,37,45,47,50,52,56^ that make them susceptible to LBP based on kinesiopathologic model of LBP^63^. Indeed, Nelson-Wong and Callaghan^64^ reported significantly higher rates of clinical LBP (requiring any kind of medical care or resulting in 3 days or more off from work, school, or recreation) over the 3-year-follow-up period and a threefold increase in the odds of experiencing an episode of clinical LBP over the first 24 months in PDs compared to NPDs. Therefore, PDs are potentially known as a pre-clinical LBP population^64^ reporting symptoms of the same quality and location during prolonged standing as those typically experienced by people with LBP^62^.

Investigations on PDs started in 2008^24,44,65^ and many studies have reported the high prevalence of standing-induced LBP and the associated risk factors in PDs ever since (31–80%)^9–61^. However, there have been contradictory findings with regard to some PDs’ characteristics in muscular, psychological, and postural variables, as well as movement patterns. For example, they have been reported to have lower endurance of side-bridge^32^ and side-lying repetitive (dominant) leg raising exercise with similar strength losses^55^. However, some studies have not reported any differences in Gluteus Medius (GMed) muscle endurance during side support^42^, or the reverse side-bridge^47^. In terms of muscle recruitment strategies, Nelson-Wong et al.^37^ reported a ‘top-down’ strategy with lumbar extensors activated prior to GMax for return-to-stand from forward flexion while two other studies found no significant difference in typical muscle recruitment strategy (bottom-up) between the two groups^13,47^.

With regard to psychological variables, Park^47^ has reported worse scores in Pain Catastrophizing Scale (PCS) for PDs in contrast to Hwang et al.^27^, and Naseri and Kahrizi^36^. Likewise, Sorensen et al.^49^ have reported no difference between the two groups but a large correlation between average standing-induced LBP intensity and PCS score (r = 0.87, *P* = 0.06) for only PDs with a maximum VAS score ≥ 20 mm (clinically important level of LBP). With regard to Fear of Pain Questionnaire (FPQ-III), Park^47^ reported worse scores in PDs. Although Hwang et al.^26^ reported no difference between PDs and NPDs in FPQ-III scores, he found out that a greater Fear of Pain minor subscale scores predicted greater initial LBP and a more rapid increase in LBP over the 2 h of standing. Likewise, Sorensen et al.^49^ reported no difference in FPQ-III scores between the two groups although they found a large and significant correlation between average standing-induced LBP intensity and FPQ-III for only PDs with a maximum VAS score ≥ 20 mm (r = 0.91, *P* = 0.03), suggesting that if pain exceeds a clinically meaningful threshold during standing, psychological status may modulate pain intensity.

In terms of postural variables, some studies have reported larger lumbar lordosis in PDs prior to prolonged standing^35,51^ and a significant relationship with Max VAS (r = 0.46, *P* = 0.02)^51^. While other studies have indicated no difference in lumbar lordosis^23,26,56^ and lumbar angle (lumbar spine with respect to pelvis)^19^ between PDs and NPDs. In addition, there are inconsistent reports on the rates of lumbar fidgets, defined as fast and large change in lumbar spine angle that quickly returns to its original orientation, between PDs and NPDs^18,19,61^. While Gallagher and Callaghan^18,19^ have reported lower frequency of lumbar spine flexion/extension fidgets in PDs, Winberg et al.^61^ have reported no significant differences in lumbar spine fidgets between PDs and NPDs.

This is also the case for AHAbd test that reliably assesses an individual’s ability to maintain trunk and pelvis alignment while moving the lower extremity in an unstable side-lying position and can discriminate between PDs and NPDs^42^. Although Nelson-Wong et al.^42^, Khoshroo et al.^29^, Homaie-Morad^25^, and Alghosi^10^ found that PDs were scored higher (worse) and that individuals with the positive test have 3.85^42^ and 18.3^29^ times higher odds of developing LBP during the prolonged standing protocol, Park^47^ reported no difference in examiner-rated AHAbd scores between the two groups.

Since PDs may be considered a pre-clinical LBP population^64^ at risk of clinical LBP development and thus exacting great social and economic costs, it is necessary to comprehensively investigate their distinctive characteristics and the risk factors of standing-induced LBP based on which appropriate preventive measures can be planned, particularly for work-related prolonged standing LBP and its associated occupational burdens^66,67^. Future researchers can also apply findings from the present study as a rationale to plan their studies regarding predictors of clinical LBP.

According to Offord and Chmura Kraemer^68^, a risk factor is a type of correlate associated with an increased probability of an unpleasant outcome, and precedes it. Therefore, PDs’ characteristics were investigated prior to (without being affected by the prolonged standing discomfort and time), during, and following prolonged standing exposure to identify risk factors occurring before pain reports in observational studies. Moreover, if a risk factor can be modified or changed through intervention, then this is termed a variable risk factor^68^. Accordingly, intervention studies were also included. To the best of our knowledge, there is no systematic review in this regard to provide a coherent and clear picture of PDs’ characteristics. Therefore, the purpose of this study was to conduct a systematic review with meta-analysis regarding prolonged standing PDs’ distinctive characteristics and the risk factors associated with standing-induced LBP.

## Methods

### Search strategy

This review was a-priori registered^69^ and executed according to the PRISMA-2020 statement guidelines (Preferred Reporting Items for Systematic Reviews and Meta-Analyses)^70^. To identify relevant publications, we systematically searched Scopus, Web of Science, and PubMed from database inception to 14 July 2022 using a combination of terms relevant to ‘standing’ and ‘low back pain development’ (Supplementary material 1). ProQuest and Google Scholar were also searched. Relevant systematic reviews and references of relevant publications were also scrutinized manually with the aim to identify additional potentially eligible literature.

### Eligibility criteria

Studies with low risk of bias in English and Persian using a methodological quality scoring system^71^ were deemed eligible for inclusion if they were laboratory studies classifying adult PDs and NPDs using prolonged standing duration greater than 42 min. This duration was based on Coenen et al.s’^72^ systematic review indicating clinically relevant levels of low back symptoms were reached after 42 min in PDs. Another requirement was asymptomatic participants could not have a history of LBP that was significant enough to disrupt their activities of daily living, or to cause the individual to seek medical care, or more than 3 days off work or school. Reviews, editorials, letters, conference proceedings, and duplicates were excluded.

### Study selection

**Figure 1:**
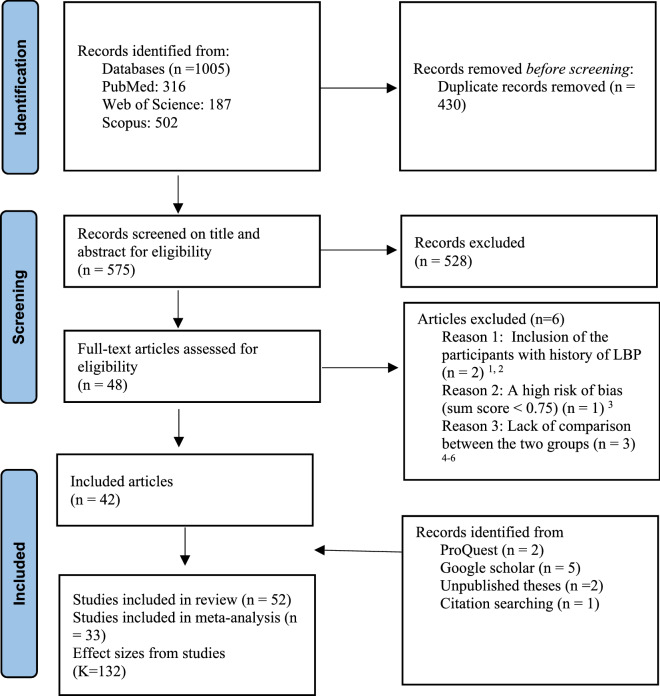
PRISMA 2020 flow diagram for included studies in the systematic review and meta-analysis.^1^Bussey, M. D.; Kennedy, J. E.; Kennedy, G., Gluteus medius coactivation response in field hockey players with and without low back pain. *Physical therapy in sport: official journal of the Association of Chartered Physiotherapists in Sports Medicine* **2016,** *17*, 24–9. ^2^Rodríguez-Romero, B.; Smith, M. D.; Quintela-Del-Rio, A.; Johnston, V., What Psychosocial and Physical Characteristics Differentiate Office Workers Who Develop Standing-Induced Low Back Pain? A Cross-Sectional Study. *International journal of environmental research and public health* **2020,** *17* (19).^3^Gregory and Callaghan^65^. ^4^Gallagher, K. M.; Wong, A.; Callaghan, J. P., Possible mechanisms for the reduction of low back pain associated with standing on a sloped surface. *Gait & posture* **2013,** *37* (3), 313–8. ^5^Nelson-Wong and Callaghan^64^. ^6^Sorensen et al.^62^.

Two reviewers (FK, MB) independently screened all potentially relevant titles and abstracts for eligibility. If necessary, full-text articles were checked for inclusion and recorded reasons for the ineligible studies. All disagreements were resolved by consensus, if not, by discussion with a third review author (FS). We recorded the selection process in sufficient details and the protocol followed the recommendations established by the PRISMA (Fig. 1).

### Data extraction and risk of bias assessment

Two authors independently reviewed the titles and abstracts of all identified studies to determine eligibility. Two reviewers (FK, MB) assessed all selected studies independently for risk of bias and relevant data extraction. In cases of disagreement, consensus was reached during a meeting. Risk of bias was evaluated using a methodological quality scoring system^71^ based on fourteen criteria for the reporting of study methods and results (Supplementary material 2). Studies scored ≥ 0.75 out of 1.00 were considered to be of high methodological quality^71^ and thus low risk of bias. The following data from each included study were extracted: authors and year of publication, study design, study population and sample size, sample description (i.e., demographics, country and other relevant specifics), standing condition (i.e., prolonged standing duration, VAS cut-off point to classify PDs and NPDs, and pain report intervals), measuring instruments, associated physiological and psychological outcomes, raw scores, and related descriptive statistics [mean, standard deviation (SD)] for pertinent measured variables/risk factors. Authors were emailed with requests to provide additional information if insufficient information was reported in the studies.

### Data synthesis and statistical analysis

All outcomes were narratively summarized and tabulated. Where two or more studies reported the same type of risk factor or measure, the results were combined and analyzed using STATA software version 17. PDs were compared with NPDs in demographics, biomechanical, and psychological outcomes. Weighted mean differences (WMD), standardized mean differences (SMD), Hedge’s g (If the sample size was less than 20 and the groups were dissimilar in size^73^) to determine the pooled effect size of each risk factor. Means and standard deviations extracted from studies were used to calculate effect sizes. Effect sizes for each continuous variable were defined as trivial (0–0.2), small (0.2–0.6), moderate (0.6–1.2), large (1.2–2.0), or very large (> 2.0)^74^. The random effects model was chosen on all outcome variables due to consensus that the random-effects model is more realistic in most situations as there exist methodological and substantive differences typically found among the combined studies in a meta-analysis^75,76^. Furthermore, inferences made from random-effects models are unconditional and may be extrapolated to a population of studies larger than the sample that were not included in the meta-analysis or that have not yet been done^76^. The Hartung–Knapp–Sidik–Jonkman (HKSJ) method for random effects meta-analysis was used for the variables as this method can better account for low statistical power when five or fewer trials are included in a meta-analysis^77^. The *P* value for significance of the pooled effect analyses was set at < 0.05. Heterogeneity was assessed using the I^2^ test and the Q test. Subgroup analysis with significant difference and substantial heterogeneity were performed based on age (> 25 vs ≤ 25 years old) and sex (males vs females). For the analysis of publication bias, Egger's test, and Duval and Tweedie’s trim and fill test were conducted to analyze asymmetrical distributions of effect sizes included in the meta-analysis.

## Results

### Study selection

The flow chart of the search and selection of literature is presented in Fig. 1. The search strategy yielded, after removing duplicates, 575 individual articles that were screened for inclusion. A total of 48 full-text articles were considered, of which 42 met the inclusion criteria. Searching ProQuest^26,60^ and Google Scholar^17,35,36,47,58^ yielded 7 more eligible studies. 2 unpublished theses were also identified at the University of Tehran^10,25^. After screening the reference lists of these articles, one more article was added^41^ resulting in a total of 46 articles and 6 theses (reporting from 42 studies three of which had recruited participants already classified by other studies excluding them, with 1070 participants 528 PDs and 542 NPDs) included in the current review from which risk of bias assessment and data-extraction was conducted (Supplementary material 3). Nineteen studies were not used in the meta-analysis^11,12,15–17,20,22,28,30,31,38,40,41,45,48,50,52,55,78^.

### Risk of bias

Only studies with low risk of bias could be included in this study. The average methodological quality score of the included studies was 0.85 (SD:0.06) out of 1, ranging from 0.77 to 0.96.

### PDs and NPDs’ personal characteristics

Table 1 summarizes the reported demographics of PDs and NPDs from all studies. A series of random-effects models were used to analyze age^9,10,13,18,25,27,29,32,33,35,36,42–44,46,47,51,54,56–58,60,61^, height^10,23,25,27,29,32,33,36,43,44,46,47,51,54,56–58,60,61^, weight^10,13,25,27,29,32,33,36,44,46,47,51,54,56–58,60,61^, BMI^9,10,13,18,25,27,29,35,36,42–44,47,51,57,60,61^, sex (Ratio of females (PDs to NPDs) to males (PDs to NPDs)^13,18,19,21,24,27,32,34,36,42,43,46,47,51,54,56–58^, and physical activity^10,18,25,27,29,42,43,51^ for PDs and NPDs. Studies had used different scales to measure physical activity such as the Minnesota Leisure-Time Physical Activities Questionnaire^18^, modified Minnesota Leisure-Time Physical Activities Questionnaire^42^, Baecke Questionnaire of Habitual Physical Activity^10,25,27,29,51^, and Occupational Sitting and Physical Activity Questionnaire^43^. Differences in personal characteristics do not seem to be significantly associated with standing-induced LBP and the ratio of female PDs to female NPDs is not significantly different from the ratio of male PDs to male NPDs (Table 2).

**Table 1:** Demographics of PDs and NPDs.

	Pain developers			Non-pain developers		
	No	Mean	SD	No	Mean	SD
Age (years)	371	24.854	4.649	393	24.652	5.308
Height (m)	305	1.704	0.097	317	1.703	0.095
Weight (Kg)	285	66.496	11.909	305	66.334	12.835
BMI (Kg/m^2^)	293	23.144	2.966	316	23.397	3.056

**Table 2:** The meta-analysis of PDs and NPDs’ personal characteristics.

Personal characteristics	Number of studies	Size (n)	Statistical method, effect size (CI 95%)	Standard error	Z value	*P* value	Q-value	Degree of freedom	I-squared	*P* value	Trim and fill imputed studies
Age	23	764	WMD 0.023 − 0.33 to 0.37	0.182	0.12	0.89	19.13	22	0	0.63	0
Height	19	622	WMD − 0.005 (− 0.017 to − 0.007)	0.006	− 0.83	0.402	20.42	18	11.85	0.31	0
Weight	18	590	WMD − 0.088 (− 2.168 to 1.992)	1.061	− 0.08	0.93	26.01	17	34.65	0.07	0
BMI	16	609	WMD − 0.35 (− 0.861 to 0.155)	0.259	− 1.3	0.173	19.96	15	24.85	0.17	5
Physical activity	8	361	Hedge’s g − 0.093 (− 0.298 to 0.113)	0.105	− 0.88	0.376	5.56	7	0	0.59	1
Ratio of females (PDs to NPDs) to males (PDs to NPDs)	18	570	Odd ratio 0.97 (0.67–1.38)	–	− 0.16	0.86	18.33	17	7.25	0.36	5

*BMI* body mass index, *CI* confidence interval, *WMD* weighted mean difference.

### PDs and NPDs’ characteristics and associations with standing-induced LBP

There were significant differences between PDs and NPDs in terms of movement patterns, muscular, postural, psychological, structural, and anthropometric variables. PDs and NPDs’ common and distinctive characteristics have been summarized in Supplementary material 4. There were also some sex/gender-based differences between female and male PDs and NPDs. Supplementary material 5 summarizes these differences. There were some inconsistencies in findings on lumbar fidgets, AHAbd test, hip abductor endurance, lumbar lordosis, muscle recruitment strategy for return-to-stand from forward flexion, and psychological variables. Therefore, meta-analyses were run in order to calculate the pooled effect sizes to resolve the incongruencies. In addition, a meta-analysis was administered on consistent GMed co-activation findings to calculate the magnitude of the effect size.

#### Muscular variables

##### Pre-standing

PDs have been indicated to have lower amplitude of muscle activation i.e. less maximum voluntary contraction (%MVC) and more between left and right sides asymmetry in the amplitude of muscle activation at the left plantar flexors^36^, a greater average number of responsive (active) extensor muscles and a greater occurrence of extensor muscle response (95–100% of trials) due to trunk perturbations^24^, and higher bilateral GMed co-activation^14^.

##### During standing

PDs were reported to have consistent higher co-activation of the bilateral GMed muscles^9,32,44^, higher levels of muscle co-activation only during the first (before subjective report of pain) and final 30 min of 2-h standing period^39^. There is also a strong negative correlation between VAS score and co-activation index for the bilateral GMed during the acute pain development (30–90 min) (r = − 0.73)^39^. In addition, PDs have higher left lumbar erector spinae and left external oblique (trunk) co-activation^44^, higher levels of trunk muscle co-activation at the beginning and end of the 2-h standing period^39^, a decrease of trunk muscle co-activation during the acute pain development (30–90 min) of 2-h standing period with a strong negative correlation with VAS score (r = − 0.92)^39^.

##### Post-standing

PDs have been shown to have inability to recover force losses after 120 min of standing after the side-lying, repetitive (dominant) leg raising exercise performed before prolonged standing^55^, a greater rate of fatigue for contralateral GMed during the side-bridge test^32^, a greater average number of responsive (active) extensor muscles and a greater occurrence of extensor muscle response (95–100% of trials) due to trunk perturbations^24^. PDs’ standing-induced LBP was also associated with the changes in anticipatory postural adjustment amplitudes post-standing during shoulder flexion (rs = 0.43, *P* = 0.002)^33^.

#### Postural variables

##### Pre-standing

PDs showed more even distribution of intervertebral angles in upright standing poses throughout their lumbar spines and proportionately less lumbar lordosis at lower levels of the lumbar spine (L5-S1) and proportionately more lordosis at upper lumbar levels (L1-L4)^56^. PDs typically showed lower lumbar spine passive stiffness standing with a lumbar spine angle further away from their passive lumbar spine neutral zone limit, compared to NPDs standing closer to their neutral zone extension limit^17^. PDs had larger pelvic incidence sacral slope^35^, and greater mean COP displacement during 60-s quiet standing^46^. PDs also demonstrated less reliance on lumbar multifidus proprioception than that of triceps surae during 60 s quiet standing (altered proprioceptive postural control strategies)^46^.

##### During standing

There has been reported a significant correlation of the horizontal distance measures between the spinous processes of the T6 and L3 over time to LBP intensity in PDs (r value range: 0.31–0.42, *P* value range: 0.01–0.04). A significant increase of thoracic extension has been observed in PDs while there is greater thoracic flexion (by approximately 8 deg) in NPDs^20^. Although Gallagher and Callaghan^19^ reported no difference in trunk angle (trunk with respect to pelvis), PDs stood 4 degrees more than NPDs away from the maximum trunk extension angle (*P* = 0.1037).

Other distinctive characteristics relate to the association of larger lumbar lordosis within PDs with a higher Max VAS pain^60^. They have less movement of their lumbar spine^20^. In addition, PDs utilized a lower range of their lumbar spine angle compared to NPDs^20^.

PDs have greater medio-lateral (ML) COP fidget frequency^21^ farther location of COP to the heel during the first 15 min compared to NPDs^21^, lower antro-posterior (AP) COP and COP ML range, lower Velocity AP and Velocity ML range^31^, and fewer large body weight transfers (30% BW) during the first 15 min of standing^18^. They also increased their large BWS frequency more consistently compared with NPDs^43^.

##### Post-standing

PDs have had lower antro-posterior median power frequency during a 2-min eyes opened constrained standing task^15^, with an increase during a 2-min eyes closed constrained standing task in contrast to no change in NPDs^15^. ML median power frequency during the eyes opened 2-min constrained standing task increased significantly for both groups, but there was a greater change in NPDs^15^. On the foam surface, PDs had an increase in COP displacement and velocity in ML direction during 60-s standing compared to NPDs and also compared to standing on the firm surface^46^. There was an increase in PDs’ COP displacement in ML direction during 60-s standing on the foam surface compared to pre-prolonged standing^46^. Greater mean COP displacement in ML direction occurred during quiet standing on the firm condition compared with standing on the foam condition in NPDs^46^. There was also lower mean velocity during standing on the firm condition in PDs compared to pre- prolonged standing (*P* = 0.051)^46^. A greater COP regularity (decreased sample entropy) has also been reported in PDs^15^.

#### Movement patterns

##### Active hip abduction

PDs move their lumbopelvic region earlier during left hip abduction than right hip abduction which is significantly correlated with average symptom intensity during standing (r = 0.46; *P* = 0.02)^50^. They have 3.85 (95% CI 1.05–19.07)^42^ or even 18.3 (95% CI, 3.674–91.22)^29^ times higher odds of developing LBP during the prolonged standing protocol with a positive test. PDs have also self-rated AHAbd as more difficult having 6.55 (95% CI 1.14–37.75) times higher odds of developing LBP during the prolonged standing protocol with a positive test^42^.

Furthermore, PDs’ reduced motor control concurrent with increase in demands either through long-term exposure or external load have been remarkable. There is lower movement smoothness in PDs exhibiting larger angular displacement arc length during AHAbd performance (worse performance) with an external weight relative to no external weight^47^.

##### Standing extension task (lean, static and return)/maximum extension

There is more co-activity in PDs’ hip extensors (GMax, long head of Biceps Femoris)^58^. Intervertebral angles have been more evenly distributed in full extension throughout lumbar spines with the lack of lower segment contributions in PDs spread throughout the upper lumbar levels (L1–L4) compared to NPDs with larger contributions from lower lumbar segments (L5–S1)^56^. There are also larger (more extended) lumbar lordosis, L1/L2 IV angle, and sacral slope in PDs^35^.

##### Functional movement screening (FMS)

PDs scored lower (worse) in the composite and individual component FMS score^29^. The optimal cutoff scores of ≤ 14 on the FMS, 2 on the push-up, and 1 on the deep squat^29^ could discriminate PDs from NPDs. Lower FMS scores was associated with an earlier onset of and higher LBP intensity during standing^29^. PDs with at least one bilateral asymmetry on the FMS had 10 times (95% CI, 2.941–34.008) and with at least two bilateral asymmetries on the FMS had 15.5 times (95% CI, 3.814–63.359) higher odds of developing LBP during prolonged standing than NPDs^29^. LBP intensity was also correlated negatively with LBP onset (rs (30) = − 0.509, *P* = 0.004) during the prolonged standing protocol^29^.

##### Maximal trunk flexion–extension exertions

NPDs performed maximal trunk flexion–extension exertions faster than PDs^47^.

##### Submaximal lumbar flexion–extension

Before standing, PDs have been indicated to have heightened relaxation response of the hip extensors (GMax muscles) during forward flexion at the trunk compared^45^, elevated erector spinae-external oblique coactivation during return-to-stand^13^, greater GMed and lumbar erector spinae muscle activity while in flexion with respect to flexion relaxation^52^, and elevated erector spinae-internal oblique coactivation with sacroiliac joint bracing during return-to stand in PDs^13^. After prolonged standing, the heightened relaxation response for the gluteus maximus muscles was still present^45^.

##### Single-leg stance

Prior to standing, PDs had lower hip abductor activity in non-dominant unilateral stance and also lower hip extensor activity with sacroiliac joint bracing compared to NPDs^13^. After prolonged standing, PDs had higher peak activation of left GMed during left single-leg standing^45^ and increased trunk extension while NPDs had increased flexion^45^. Also, PDs had increased global pelvis lateral bend while NPDs had decreased global pelvis lateral bend during left single-leg standing^45^.

##### Stair descent

PDs had larger lumbar lordosis during stair descent^35^.

##### Stair climbing

PDs had larger lumbar lordosis, and sacral slope^35^.

##### Sitting

There was no large difference in lumbar spine flexion range of motion between sitting and standing in PDs^20^ and they demonstrated similar median thoracic angles with the angle increasing by approximately 10 degrees of greater flexion in sitting compared to standing, while the non-PDs remained consistent even when entering into sitting^20^. PDs had more extended lumbar spines and L1/L2 intervertebral angle^35^.

* Leaning forward while sitting* PDs had more prominent flexion of LL, LSL and L5/S1 IV angles compared to upright standing posture^35^.

#### Structural variable

There existed difference in the individual lumbar intervertebral disk morphology between PDs and NPD both in supine and standing position using radiography, i.e. smaller A/P ratios of each lumbar intervertebral disk (less wedging) in female PDs and male NPDs than female NPDs and male PDs^60^.

#### Anthropometric variables

A greater trunk to height ratio predicted a greater and faster increase in LBP intensities over the 2 h of standing^26^. Even with the recruiting criteria of BMI < 30 kg/m^2^, the larger BMI has been able to predict a greater severity of the pain symptoms^60^. Also, higher amounts of pain and erector spinae co-activation at the upper, middle, and low back in female PDs with large breast size has been reported^28^.

#### Meta-analysis results for biomechanical outcomes

##### Lumbar fidgets

Three papers^18,19,61^ involving 31 PDs and 34 NPDs reported results of the lumbar fidgets defined as fast and large change in lumbar spine angle that quickly returns to its original orientation based on previous work by Duarte and Zatsiorsky^79^. Using motion analysis system, it was quantified as a change of angles about the lumbar spine flexion–extension, lateral bend, and axial twist axes^18^ or the sagittal plane only^19^ in threshold of ± 3SD, window length of 50 s^18^ or 60 s^19,61^, and maximum duration of 4 s tabulated over a 15-min block expressed as a frequency per 15 min based on Duarte and Zatsiorsky^79^. Gallagher and Callaghan^18^ reported the frequency of the lumbar fidgets only for the first 15-min block and the other two^19,61^ have reported the mean frequency for each 15-min block. Lumbar fidget was found to be significantly associated with standing-induced LBP (Hedge’s g -0.72, 95% CI − 1.35 to − 0.08, *P* = 0.03, I^2^ = 38.33%, *P* = 0.24). The meta-analysis indicates that fewer lumbar fidgets while standing is associated with increased risk of standing-induced LBP.

##### Lumbar lordosis

Five studies have examined lumbar lordosis prior to prolonged standing in PDs and NPDs yielding contradictory results^23,26,35,51,56^. Two have measured lumbar curvature angle (LCA) using a motion analysis system^26,51^. The sagittal LCA was calculated by (1) finding the distance of a vector (l) from L1 to L5, (2) finding the distance of the vector (d) that is perpendicular from l to L3, (3) using the formula: 2arctan (0.5 l/d) in these studies. Values less than 180° indicated lumbar extension during standing and values greater than 180° indicated lumbar flexion during standing. Hwang^26^ found no significant difference between the two groups contrary to Sorensen et al.^51^. The results of meta-analysis did not indicate a significant difference in LCA between 57 PDs and 71 NPDs (WMD 1.45, 95% CI − 4.08 to 6.98, *P* = 0.51).

Three studies measured lumbar lordosis using radiography to calculate Cobb angle^23,35,56^ involving 37 PDs and 37 NPDs. One study^23^ had not provided means and standard deviations of the groups and the effect size was calculated using *P* value and sample size. The results did not indicate a significant difference in cobb angle between PDs and NPDs (WMD 6.91, 95% CI − 4.72 to 18.54, *P* = 0.24, I^2^ = 92.89). The Q-test and I^2^ test results indicated significant heterogeneity across studies. In order to investigate the sources of heterogeneity, subgroup analysis was performed based on age in 2 categories of average age of 25 and under, and over 25, using mixed effect analysis. The results suggested that age modified the association of the lumbar lordosis with standing-induced LBP and could successfully explain the source of heterogeneity (*P* < 0.001). As per results, with older age^35^, the lumbar lordosis differs significantly between PDs and NPDs and can better predict standing-induced LBP (WMD 18.1, 95% CI 14 to 22.2, *P* < 0.001) than in younger PDs and NPDs whose lumbar lordosis does not differ significantly^23,56^ (WMD 1.91, 95% CI − 3.03 to 6.85, *P* = 0.23).

The results of the meta-analysis for all five studies with 94 PDs and 108 NPDs also did not show a significant difference in lumbar lordosis between PDs and NPDs (Hedge’s g 0.72, 95% CI − 0.32 to 1.76, *P* = 0.15, I^2^ = 91.39%, *P* < 0.001). The Q-test and I^2^ test results indicated significant heterogeneity across studies. In order to investigate sources of heterogeneity, subgroup analysis was performed again based on age in 2 categories of average age of 25 and under, and over 25, using mixed effects analysis. The results suggested that age modified the association of the lumbar lordosis with standing-induced LBP and could successfully explain the source of heterogeneity (*P* < 0.001). As per results, with older age^35^, the lumbar lordosis differs significantly between PDs and NPDs and can better predict standing-induced LBP (Hedge’s g 2.75, 95% CI 1.89–3.61, *P* < 0.001) than in younger PDs and NPDs whose lumbar lordosis does not differ significantly^23,26,51,56^ (Hedge’s g 0.22, 95% CI − 0.29 to 0.72, *P* = 0.28).

##### AHAbd test

5 papers^10,25,29,42,47^ involving 95 PDs and 113 NPDs reported results of AHAbd test reliably assessing an individual’s ability to maintain trunk and pelvis alignment while moving the lower extremity in an unstable side-lying position and can discriminate between PDs and NPDs^42^. The test is performed with lower limbs fully extended and aligned with the trunk and head during movement. The pelvis should remain perpendicular to the supporting surface. The AHAbd is scored from 0 to 3 with higher scores reflecting a higher susceptibility to standing-induced LBP. Both sides are tested and the score from the worse of the 2 sides is assumed as the final score. Individuals are assigned as PDs if they are scored 2 or 3 in this test^42^. AHAbd test was found to be significantly associated with standing-induced LBP (WMD 0.7, 95% CI 0.36–1.05, *P* < 0.001, I^2^ = 78.63%, *P* = 0.07). The meta-analysis indicates that higher scores on the test, indicating worse motor control, is associated with increased risk of standing-induced LBP. In order to investigate sources of heterogeneity, subgroup analysis was performed based on sex/gender in three categories of male, female, and both genders, using mixed effects analysis. The results suggested that sex/gender modified the association of the AHAbd scores with standing-induced LBP and could successfully explain the source of heterogeneity (*P* < 0.001). As per results, the higher scores in males^10^ can better predict standing-induced LBP (WMD 1.177, 95% CI − 0.9 to 1.45, *P* < 0.001) than females^25,29^ (WMD, 0.68, 95% CI − 0.44 to 0.92).

##### Muscle recruitment strategy for return-to-stand from forward flexion

Three papers^13,37,47^ involving 48 PDs and 65 NPDs reported the results of the phase lag between lumbar extensors and GMax corresponding to the maximum cross-correlation calculated. Nelson-Wong et al.^37^ reported a ‘top-down’ muscle recruitment strategy with lumbar extensors activated prior to GMax for return-to-stand from forward flexion while two other studies found no significant difference in typical muscle recruitment strategy (bottom-up) between the two groups^13,47^. The results of the meta-analysis indicated no significant difference between the two groups (WMD 0.07, 95% CI − 0.04 to 0.19, *P* = 0.21, I^2^ = 76.55%, *P* = 0.21).

##### Hip abductor endurance

Four studies^32,42,47,55^ involving 67 PDs and 79 NPDs reported results of the hip abductor endurance. Viggiani and Callaghan^55^ used side-lying, repetitive (dominant) leg raising exercise. Participants had to abduct their leg for 1 s and lower the leg back down for 1 s with a metronome. After completing five repetitions (10 s of work), they were given a 5-s rest. This 2:1 work-rest ratio was considered a duty cycle. Participants were stopped if either two consecutive or five non-consecutive duty cycles were unsuccessful based on postural compensations occurring during that duty cycle. Other studies considered holding time during side bridge^32,42^, and reverse side-bridge^47^ to calculate hip abductor endurance. Hip abductor endurance was not significantly different between PDs and NPDs, and thus not associated with standing-induced LBP (Hedge’s g − 0.37, 95% CI − 0.88 to 0.14, *P* = 0.15, I^2^ = 56.66).

##### GMed co-activation

Six articles investigated GMed co-activation in PDs and NPDs during prolonged standing; however, only three of them^9,32,44^ were included in the analysis. One study was not included as it had calculated co-activation during 5 min^14^ and one had sitting intervals during prolonged standing^34^ and one study had not reported the necessary data and did not have the data available to provide^39^. From the included studies, one used co-activation index^9^ and two others crossed-correlation^32,44^ to calculate co-activation. In 42 PDs and 20 NPDs, higher GMed co-activation was significantly associated with development of standing-induced LBP (Hedge’s g 4.24, 95% CI 3.18–5.3, *P* < 0.001, I^2^ = 25.13%, *P* = 0.39).

#### Meta-analysis results for psychological outcomes

4 studies have investigated the PCS^27,36,47,49^ involving 84 PDs and 108 NPDs. It is a 13-item questionnaire. The PCS measures the extent of catastrophic thoughts a person reports in response to pain^80^.The scores range from 0 to 52. Higher scores on the PCS indicate a higher degree of catastrophizing. The PCS has been shown to be reliable and valid in clinical and healthy populations^80^. PDs seem to get worse scores in this scale (WMD 2.85, 95% CI 0.51–5.19, *P* = 0.02, I^2^ = 27.95%, *P* = 0.52). The meta-analysis shows catastrophic thoughts a person reports in response to pain may be significantly associated with standing-induced LBP.

3 studies have investigated the FPQ-III involving 74 PDs and 93 NPDs. It is a 30-item questionnaire that measures fear of pain associated with specific events^81^. The total range of scores is from 30 to 150. Higher scores on the FPQ-III indicate higher fear of pain. The FPQ-III has been shown to be reliable and valid in clinical^81^ and healthy populations^82^. Park^47^ reported worse scores in PDs. Hwang^26^ and Sorensen et al.^49^ reported no significant difference between PDs and NPDs in FPQ-III scores. The results of the meta-analysis indicated that FPQ-III score was not significantly different between PDs and NPDs, and thus not associated with standing-induced LBP (WMD 5.31, 95% CI − 3.6 to 14.23, *P* = 0.24, I^2^ = 57.66%, *P* = 0.1).

#### Analyses of publication bias

In order to analyze the file-drawer-problem impact on the meta-analyses, Duval and Tweedie’s trim and fill test^83^ and Egger’s test were conducted as the number of studies assessing each of the above-mentioned variables was less than 10 and thus funnel plot could not be used^84^. First, Duval and Tweedie’s trim and fill test was conducted^83^. If there was no publication bias present, the studies included in the analysis would be symmetrically distributed on the funnel plot. The Duval and Tweedie’s trim and fill method imputed zero potential missing studies for AHAbd, GMed co-activation during prolonged standing, radiographic lumbar Cobb angle prior to prolonged standing, lumbar lordosis prior to prolonged standing, phase lag between lumbar extensors and GMax for return to extension from full lumbar flexion, PCS, FPQ-III, and lumbar fidgets. One potential missing study was imputed for the hip abductor endurance that slightly changed the results. Also, one potential missing study was imputed for lumbar lordosis which substantially changed the results. The results of Egger’s test indicated publication bias for hip abductor endurance (Egger’s − 6.81, *P* = 0.03) and PCS (Egger’s 8.82, *P* = 0.06).

#### The effectiveness of intervention for standing-induced LBP

In order to find the risk factors, intervention studies were also examined to find out which variables would change as a result of an intervention^68^ leading to significantly reduced pain reports. Supplementary material 6 summarizes the results of intervention studies. All interventions have been effective to either significantly decrease subjective pain level by providing temporary or lasting recovery, or decrease the number of PDs. Lee et al.^30^ reported that using footrest, in a 2-h protocol having the right foot and then the left foot raised on the footrest, and then both on the floor each for 5 min, could not significantly decrease LBD. However, LBD reported on two 2-h standing with and without a footrest appeared to be diverging at 2 h (51.1 mm (± 24.2 mm) without a footrest and 32.0 mm (± 15.2 mm) with the footrest). LBD seemed to increase more rapidly in the final 30 min in the no footrest condition. In contrast, Fewster et al.^16^ reported that using footrest in an 80-min 3–1–3–1 protocol cycling through level ground standing and one-min leg raise resulted in re-classification of almost all previously categorized PDs as NPDs (except one PD).

2-h and 1-h of standing on an anti-fatigue mat has been reported to significantly decrease subjective pain level^9,61^. However, it failed to significantly decrease the number of PDs^9^.

Using 3-min vibration every 12 min during 2-h standing and 3-min vibration applied at the 2-h and 2.25-h marks during a 2.5-h standing task, Cardenas and Gregory^12^ and Lurie et al.^31^ reported that LBP prior to each vibration bout was significantly higher than that immediately following each vibration bout, suggesting a temporary relief of pain in PDs. However, LBP returned to pre-vibration levels when the vibration ceased^31^ and the level of perceived LBP at the end of the 2 h on the control day was not significantly different from that on the vibration day^12^.

Side-lying, repetitive (dominant) leg raising exercise before prolonged standing could lead to lower peak VAS scores during the fatigue session. Female reductions began after 90 min and male reductions began after 120 min^54^.

75-min prolonged standing on declining sloped surface of 16 degrees reduced PDs’ LBP scores by 58% compared to level ground^19^. Self-selected alternation between standing on a 16 degrees incline and decline surface also could reduce LBP scores by 59.4% for PD when compared to level standing^40^.

Bending forward to full spine flexion for 5 s every 15 min at the start and during 2-h standing decreased LBP by 36% (10 mm) at the end of a 2-h standing and at 75 min onward, post-flexion LBP scores became significantly lower than control day flexion scores. After the full 120 min, pre-flexion LBP scores became significantly lower than control day scores^52^.

Using sit-stand desk, all PDs in intervention (12-week graded standing exposure using a sit-stand desk and home exercise) and control group (sit-stand desk without instruction or home exercise) had decreased LBP regardless of intervention (Day 1 average VAS = 10.43 ± 1.65 mm, Day 2 average VAS = 2.3 ± 4.0 mm, Day 1 maximum VAS = 21.9 ± 3.73 mm, Day 2 maximum VAS = 5.1 ± 6.9 mm)^43^. Also, a 124-min of standing work with seated breaks at a 3:1 stand-sit ratio with increasing durations from 3:1 min to 48:16 min could successfully reduce LBPs in PDs to the level of NPDs’, with mean pain scores of 13 mm which was only slightly higher than the clinical LBP threshold of 10 mm^34^. However, in a 3:1 stand to sit ratio during two 1-h blocks (45-min standing followed by a 15-min sitting), when PDs transitioned to a seated break, they had an average decrease of 12.5 mm (7 mm) in their subjective LBP reports and upon standing again demonstrated an increase of 16.4 mm (9.2 mm) over the 45 min to even a higher level than was present in the first 45 min of standing^20^. Likewise, two seated breaks over 4.5 h (110-min standing followed by 35-min sitting, 110-min standing followed by 10-min sitting, and 55-min standing) reduced LBP to a median of zero Levels of discomfort intensity, however, LBP increased again up to a median of two at the end of each of standing exposure^59^.

After 4-week progressive stabilization-based exercise program PDs in the intervention group had significantly lower VAS scores (8.93 ± 3.66 mm) than PDs of the control group (16.5 ± 6.3 mm)^38^.

5-min walking breaks every 25 min during 2-h standing led to re-categorization of 70% of PDs as NPDs during 2-h standing with walking breaks (22.1 (10.8) mm compared to 6.4 (1.8) mm)^22^.

## Discussion

We intended to characterize PDs’ distinctive characteristics and describe the evidence on acute associations and risk factors of standing-induced LBP from the laboratory studies. Significant differences between PDs and NPDs in terms of movement patterns, muscular, postural, psychological, structural, and anthropometric variables have been reported. Some of these differences have been shown to be resolved concurrent with the significant reduction of standing-induced LBP using following interventions which provides some insights on possible risk factors.

According to the definition of a risk factor^68^, GMed co-activation is a correlate of standing-induced LBP^39^ that has been shown to exist prior to prolonged standing exposure^14^ and consistently during standing^9,32,44^ and before pain emergence^39^. From the intervention that could successfully result in lasting decrease of standing-induced LBP, although PDs’ usual GMed activation pattern has changed concurrently with pain reduction in some interventions^16,40,54^, in some other intervention studies, usual GMed co-activation pattern related to LBP remained unchanged irrespective of significant reductions in standing-induced LBP^9,34,38^ highlighting the possible hypothesis that the increase in muscle co-activation may serve as a compensatory mechanism for poor postural control during level standing, leading to pain in areas of the low back^16^. On the whole, the findings support the notion that LBP development is not simply a response of GMed muscle activation patterns. Besides, the unchanged PDs’ usual GMed co-activation pattern related to LBP in successful intervention studies^9,34,38^ may highlight the importance of underlying postural mechanism and postural variation.

In general, the meta-analysis result showed no significant difference in lumbar lordosis between the two groups. One reason for this insignificant effect size may relate to the more even distribution of intervertebral angles in upright standing poses throughout PDs’ lumbar spines, proportionately less lumbar lordosis at lower levels (L5–S1) of the lumbar spine and proportionately more lordosis at upper lumbar levels (L1–L4) in PDs^56^, thus a total lumbar lordosis cannot be an appropriate measure to discriminate between PDs and NPDs. It should be noted that not only in upright standing pose, but also in full extension more even distribution of PDs’ intervertebral angles exists throughout lumbar spine with the lack of lower segment contributions spread throughout the upper lumbar levels (L1–L4) compared to NPDs with larger contributions from lower lumbar segments (L5–S1)^56^. On the other hand, according to Sahrmann’s “path of least resistance” concept, the movement occurs in a direction with the least resistance where the sustained posture and repeated movements happen^85^. PDs’ lumbar spine changes (significantly larger lordosis) were more prominent towards extension in their movement patterns as well. For example, in single leg stance, PDs had significantly increased trunk extension^45^. In addition, one study showed that PDs had larger lumbar lordosis than NPDs in stair climbing, standing maximum extension, and stair decent (Hedge’s g 3.07, 2.41, and 1.27, respectively)^35^.

It should also be noted that there is a significant correlation of the horizontal distance measures between the spinous processes of the T6 and L3 over time to LBP intensity in PDs (r value range: 0.31–0.42, *P* value range: 0.01–0.04) highlighting the importance of considering not only lumbar segment, but also other segment such as thoracic spine^26^. This significant relationship may be due to tendency towards either the increase of upper lumbar lordosis or thoracic flexion. It is in line with Viggiani et al.^56^ who have reported proportionately more lordosis at higher lumbar levels. However, significant increase of thoracic extension have been evidenced in PDs while there is greater thoracic flexion (by approximately 8 deg) in NPDs^20^.

In many interventional studies, significant decrease of standing-induced LBP was concomitant with changes in posture. Using footrest increased lumbar spine flexion in comparison to level standing during both the intervals of elevated leg standing and even level standing over time^16^ and could maintain UL lordosis as it moved away from usual standing posture without a footrest and became slightly less lordotic^30^. Furthermore, using side-lying, repetitive (dominant) leg raising exercise before prolonged standing, Viggiani and Callaghan^54^ reported that females’ reductions began after 90 min while males’ reductions began after 120 min. Interestingly, in contrast to male PDs exhibiting more anterior pelvic tilt and lumbar extension, female PDs had more posterior pelvic tilt and lumbar flexion than female NPDs during the fatigue session compared to the control session that is in line with other successful intervention to reduce pain causing more lumbar flexion. Besides, spine posture measures were similar between PDs and non-PDs in a 124-min of standing work with seated breaks^34^ highlighting the similarity in posture as a possible reason for the significant reduction of pain.

The meta-analysis results of the AHAbd test shows that higher scores on the test, indicating worse motor control, is associated with increased risk of standing-induced LBP (WMD 0.7, 95% CI 0.36–1.05, *P* < 0.001). Moreover, calculating the correlation between average standing-induced LBP and AHAbd test using the available raw data from one study^69^ indicated a significant correlation result (rs = 0.41, *P* = 0.001). Therefore, altered motor control displayed in AHAbd test can be considered a probable risk factor for standing-induced LBP based on the definition of risk factor presented by Offord and Chmura Kraemer^68^.

Other PD’s distinctive characteristics such as less movement of lumbar spine^20^ and lower frequency of lumbar fidgets^18,19^ and a lower range of lumbar spine angle compared to NPDs^20^ seem to change concurrently with the significant reduction of standing-induced LBP using various interventions. During 75-min prolonged standing on declining sloped surface of 16°, participants performed more fidgets over the standing exposure^19^. Nelson-Wong and Callaghan^40^ also reported that self-selected alternation between standing on a 16° incline and decline surface created a favorable minimal postural variability in both pelvic and lumbar spine angles concurrent with pain reduction^40^. Interestingly, Gallagher et al.^20^ with their 3:1 stand to sit ratio during two 1-h blocks (45-min standing followed by a 15-min sitting) which had reported a 15-min seated break failed to provide lasting recovery of LBP from standing and pain increased to a an even higher level after resuming standing, had observed PDs moved through a limited range of their lumbar spine angle and increased thoracic extension resulting in static postures with no significant difference in the range of motion of their lumbar spine flexion between sitting and standing that means adopting a less dynamic posture than NPDs may have led to failure to lasting decrease of pain . Likewise, in the protocol of 4.5 h over three periods with two seated breaks with significant increase of pain after resuming standing, PDs demonstrated a statistically significant lower medio-lateral pelvic movement in the progression of the three standing exposures than NPDs (less dynamic strategy)^59^. Furthermore, using 5-min walking breaks every 25 min during 2-h standing, no kinematic differences were observed between PDs and NPDs. Median lumbar flexion and lumbar region range of motion in the coronal and transverse planes increased during walking compared to standing for both groups (more dynamic strategy)^22^.

PDs have also been reported to have less large BWS (30% BW) during the first 15 min^18^. Concurrent with significant pain reduction, 12-week graded standing exposure using a sit-stand desk and home exercise for the intervention group and only sit-stand desk without instruction or home exercise for the control group, made all PDs adopt a more dynamic strategy with significantly increased large BWS frequency after 12 weeks^43^. Likewise, anti-fatigue mat tended to increase the number of BWS by 116% in NPDs and 54% in PDs^61^ and side-lying, repetitive (dominant) leg raising exercise before prolonged standing increased the small and large BWTs with time during the 30-min block for all participants with a similar trend extending to the 45-min block^54^.

PDs indicated farther location of COP to the heel during the first 15 min compared to NPDs^21^. During successful interventional study of 75-min prolonged standing on declining sloped surface, trunk center of gravity moved posteriorly and vertically aligned with the right ankle joint for all participants compared to level ground standing where PDs had farther location of COP to the heel during the first 15 min compared to NPDs^21^.

PDs also indicated lower COP AP and COP ML range, lower velocity AP and velocity ML range^31^. The anti-fatigue mat (60-min of standing) increased total COP movement, with both PDs and NPDs exhibiting greater COP excursions on the anti-fatigue mat compared to the rigid floor condition (NPD 55% increase; PD 35% increase). Aligned with the findings of the above-mentioned study, using side-lying, repetitive (dominant) leg raising exercise before prolonged standing could increase COP movement responses in all participants^54^.

Finally, psychological variables should cautiously be considered as a risk factor at present based on the included studies. Although meta-analysis results of the PCS indicated significantly worse scores in PDs (WMD 2.85, 95% CI 0.51–5.19, *P* = 0.02), it is not clear how significant an average higher score of 2.85 out 52 in PDs can be in reality due to lack of reports on minimal detectable change for PD population. Particularly, only one out of 4 studies has reported a significant difference in PCS between the two groups^47^. In addition to PCS, the results of the meta-analysis indicated that FPQ-III score was not significantly different between PDs and NPDs.

### Implications for practice

Our findings provide some guidance on identifying PDs as AHAbd test and GMed co-activation were found to be significantly associated with standing-induced LBP. The meta-analysis results indicated that higher scores of the AHAbd test, or rather less PDs’ motor control and higher GMed co-activation during standing are associated with increased risk of standing-induced LBP. Based on evidence examined in this review, PDs’ movement patterns, muscular, postural, psychological, structural, and anthropometric aspects need to be considered in order to design an appropriate preventive program. Especially, it can be concluded that interventions to prevent standing-induced LBP should mostly aim at postural underlying mechanism and its variations during standing as their manipulation through various interventions could successfully lead to the reduction of standing-induced LBP.

### Methodological considerations

First, there is inconsistency in the definition of whom is considered a PD. Although most of studies have this inclusion criterion that participants should not have any lifetime event of LBP that was significant enough to seek medical care, or that resulted in greater than 3 days off work or school, some studies have only limited the history of LBP over the previous 12 months^11,12,24,28,34,44,52^ and their participants may have had the history of LBP in their lifetime or even pain in other body regions including upper or middle back that may have had an effect on their results as a confounding factor. Hence, the results of such studies should cautiously be extrapolated. Secondly, this confounding factor should be considered in future studies.

Different thresholds have been used in studies to dichotomize PDs and NPDs such as any VAS value above 0 mm and remaining above 0 mm at all subsequent time points during standing^26,27,60^, any symptoms after baseline and maintaining the symptoms throughout prolonged standing^28,49–51^, a low back VAS score of at least 8 mm at any point during prolonged standing^52^, the maximum VAS of more than 10 mm during prolonged standing^9,10,24,25,33^, a low back VAS score of at least 10 mm at any point^12,20,29,31,36,37,45,47^ from baseline^57,61^ during prolonged standing, an increase of low back discomfort at least 1 level of LBD intensity in numeric rating scale during exposure periods^59^, an increase in VAS scores of 10 mm at any point^34,42,58^ from baseline^18,19,22,23,35,41,43,54,55^, any change in VAS score greater than 10 mm from baseline during the prolonged standing^14–16,21,30,32,38–40,46,48^, 2 consecutive VAS scores 10 mm greater than the baseline^13^, and a VAS rating greater than 20 mm at any point during the study, and also an overall average VAS rating greater than 10 mm^44^. One study stopped prolonged standing as soon as pain reported was equal to or greater than 10 VAS score at any point during standing^57^. More researches are needed to set a fixed cut-off point to discriminate PDs.

Studies have mostly assessed low back pain. Only five studies have assessed discomfort^11,24,30,44,59^. It is highly recommended that the future researches not only use VAS reports, but also record pain symptoms as Gallagher et al.^86^ investigating relationship between qualitative and quantitative measures of pain development during prolonged standing induced LBP development using Short-Form McGill Pain Questionnaire found out that Pain symptom were reported 31.3 (± 24.8) minutes earlier than the VAS reports. Eight participants (44% of all NPDs) were NPDs with the VAS and PDs with the symptom method based on three consecutive pain symptom reports every 7.5-min (*P* = 0.0047). On the other hand, the relatively high rates of clinical LBP in NPDs (34.6%) based on VAS reports during the 3-year follow-up by Nelson-Wong et al.^64^ further verifies the importance of using pain symptom reports to identify PDs as they may have been mis-classified as NPDs using merely VAS reports.

Pain and discomfort have usually been reported every 15 min during prolonged standing. However, some studies have used different intervals such as 5 min^20^, 7.5 min^18,19,23,35,46^, 30 min^30^. Some studies have reported magnitudes of the area within which participants have performed prolonged standing such as 64 × 52 cm^9^, 0.50 × 0.46 m^10,11,24,25,32,44,47^, 0.61 × 1.22 m^50^, a 2-foot square area^13^, and 0.5 × 0.48 m^33,48^. However, it is necessary to introduce one standard protocol for the researches in this field.

Studies have used different standing duration exposure from 2-h prolonged standing protocol such as 75-min^19^, 2.5-h^31^, 1-h^46,61^, 80-min^13^, 4.5 h over three periods with two seated breaks (110-min standing followed by 35-min sitting, 110-min standing followed by 10-min sitting, and 55-min standing)^59^, a 3:1 stand to sit ratio during two 1-h blocks (45-min standing followed by a 15-min sitting)^20^. In a systematic review pooled dose–response associations indicated that clinically relevant levels of low back symptoms emerged after 42 min in PDs^87^. Based on this finding, it is recommended to design a standard prolonged standing protocol that all researches can use a unified procedure.

There are variations in the experimental setup. Most studies have used active tasks involving upper extremities during level standing. However, some have used different tasks without moving upper extremities such as quite reading^13^, watching^22,46^, and self-directed computer activities such as reading documents and internet browsing^30^. It should be noted that task type is of great importance as Nelson-Wong and Callaghan^40^ reported the effect of task on GMed and trunk co-activation that assembly and sorting tasks had increased CCI values similarly while the boredom task (without moving upper extremities) had elicited significantly lower co-activation at the trunk and GMed than more active tasks. PDs have been evidenced to have difficulty in maintaining trunk and pelvis alignment while moving the lower extremity in an unstable position during AHAbd test with a low-level demand^10,25,29,42^ which is indicative of altered trunk stability in PDs. On the other hand, the success of 4-week progressive stabilization-based exercise program to significantly reduce LBP development in PDs^38^ and recruiting trunk surface muscle co-activation in tasks such as return-to-stand in PDs^13^ further verify impaired trunk stability in these people. Therefore, using active tasks involving upper extremities during prolonged standing is recommended to better elicit pain and reveal their distinctive characteristics.

Substantial evidence on PDs’ distinctive features thorough laboratory studies was found, with data on physiological outcomes providing insight into possible mechanisms for LBP development. Although the evidence presented in this review provides detailed information about PDs’ distinctive acute response to prolonged standing in a controlled situation, information on responses to prolonged standing outside a laboratory setting is lacking. Besides, the included studies were typically conducted among groups of relatively healthy university populations. Therefore, the findings cannot readily be generalized to other populations with comorbidities such as pre-existing musculoskeletal symptoms.

Most of studies have recruited participants with the age range of 18–35. However, a few studies have used wider age range^43,59^. Although Wall et al.^59^ recruiting participants within 18–67 years found no significant effect of age on standing-induced LBP and its associations, it is recommended to further investigate such issues in different age ranges as our results indicated that age could modify the association of the lumbar lordosis with standing-induced LBP. There may be such moderating effect of age on other variables as well.

Approximately one third of studies excluded participants from employment in a task that required prolonged static standing during the past 12 months^10,13–15,17–22,25,26,29,35–42,45–47,49–51,54,55,60^. Thus, the findings from this review should be cautiously be generalized to individuals habituated to prolonged standing. Besides, many studies scored relatively low on the sample size, sufficient characterization of PDs and NPDs (characterization by gender instead), report on confidence intervals or standard errors, and control of confounding variables in results such as gender. Therefore, such aspects should deserve more attention in future researches as they may underlie some inconsistencies in findings.

## Conclusion

We intended to characterize PDs’ distinctive features and describe the evidence on acute associations and predictors of standing-induced LBP from the laboratory studies. Significant differences between PDs and NPDs in terms of movement patterns, muscular, postural, psychological, structural, and anthropometric variables have been evidenced. Some of these differences have been shown to be manipulated using interventions. Various interventions have consistently shown that postural variables and postural variations during standing seem to play a significant role in standing-induced LBP reduction. Based on the definition by Offord and Chmura Kraemer^68^ that a risk factor is a type of correlate associated with an increased probability of an unpleasant outcome and precedes it, altered motor control displayed in AHAbd test and higher lumbar lordosis in individuals over 25 years seem to be probable risk factors for standing-induced LBP. In order to detect standing-induced risk factors, future researchers should investigate the association of the reported distinctive characteristics to the standing-induced LBP and that whether they are manipulable through various interventions.

## Supplementary Information

Supplementary Information.

## Data Availability

The datasets used and/or analyzed during the current study are available from the corresponding author on reasonable request.

## Abbreviations

AHAbd: Active hip abduction
AP: Antero-posterior
BMI: Body mass index
BWS: Body weight shift
CI: Confidence interval
COP: Center of pressure
FMS: Functional movement screening
FPQ: Fear of Pain Questionnaire
GMax: Gluteus maximus
Gmed: Gluteus medius
LBP: Low back pain
LCA: Lumbar curvature angle
Gmax: Gluteus maximum
MDC: Minimal detectable change
ML: Medio-lateral
NPD: Non-pain developer
OR: Odd ratio
PCS: Pain Catastrophizing Scale
PD: Pain developer
VAS: Visual Analogue Scale
WMD: Weighted mean difference
