# Supplementary material for: Distinctive characteristics of prolonged standing low back pain developers’ and the associated risk factors: systematic review and meta-analysis
Source: Sci Rep. 2023 Apr 19;13:6392. doi: 10.1038/s41598-023-33590-5 (PMC10115839; doi:10.1038/s41598-023-33590-5)
Supplement: Supplementary file 1 — Supplementary Information. [file 41598_2023_33590_MOESM1_ESM.docx]

**Supplementary Material 1**

PubMed search strategy

## ("active hip abduction"[Text Word] OR "long term standing"[Text Word] OR "long term standing"[Text Word] OR "Prolonged standing"[Text Word] OR "Sustained standing"[Text Word] OR "Continuous standing"[Text Word] OR "2 h standing"[Text Word] OR "2 hour trial*"[Text Word] OR "2 h"[Title/Abstract] OR "2 hours"[Title/Abstract] OR "Prolonged standing"[Title/Abstract] OR "standing exposure"[Title/Abstract]) AND ("low back pain"[MeSH Terms] OR "LBP"[Title/Abstract] OR "back pain"[Title/Abstract] OR "low back pain"[Title/Abstract] OR "Perceived pain"[Text Word] OR "Subjective discomfort"[Text Word] OR "transient LBP"[Text Word] OR "standing induced LBP"[Text Word] OR "induced pain paradigm"[Text Word] OR "induced LBP"[Text Word] OR "low back discomfort"[Title/Abstract] OR "low back discomfort"[Text Word] OR ("pain developer*"[Title/Abstract] OR "pain developer*"[Text Word] OR "standing intolerant*"[Text Word] OR "standing intolerant*"[Title/Abstract] OR ("lbdiscomfort"[All Fields] AND "developer*"[All Fields]) OR (("Low"[All Fields] AND ("back"[MeSH Terms] OR "back"[All Fields]) AND ("discomfort"[All Fields] OR "discomforting"[All Fields] OR "discomforts"[All Fields]) AND "developer*"[All Fields]) AND "title abstract"[All Fields]))) AND ("risk factor*"[Title/Abstract] OR "risk factor*"[Text Word] OR "contributing factor*"[Text Word] OR "contributing factor*"[Title/Abstract] OR "predisposing factor*"[Title/Abstract] OR "predisposing factor*"[Text Word] OR "posture"[Title/Abstract] OR "posture"[Text Word] OR "muscle activation"[Text Word] OR "muscle activation"[Title/Abstract] OR "co-activation"[Title/Abstract] OR "co-activation"[Text Word] OR "muscle activity"[Text Word] OR "muscle activity"[Title/Abstract] OR "recruitment patterns"[Title/Abstract] OR "recruitment patterns"[Text Word] OR "motor control"[Text Word] OR "motor control"[Title/Abstract] OR "instability"[Title/Abstract] OR "instability"[Text Word] OR "alignment"[Title/Abstract] OR "alignment"[Text Word] OR "malalignment"[Text Word] OR "malalignment"[Title/Abstract] OR "fatigue"[Title/Abstract] OR "fatigue"[Text Word] OR "kinematics"[Text Word] OR "kinematics"[Title/Abstract] OR "neuromuscular control"[Title/Abstract] OR "neuromuscular control"[Text Word] OR "asymmetry"[Text Word] OR "asymmetry"[Title/Abstract] OR "coordination"[Title/Abstract] OR "coordination"[Text Word] OR "lumbar posture"[Text Word] OR "lumbar posture"[Title/Abstract] OR "lumbosacral posture"[Text Word] OR "lumbosacral posture"[Title/Abstract] OR "lordosis"[Title/Abstract] OR "lordosis"[Text Word] OR "hyperlordosis"[Text Word] OR "hypolordosis"[Text Word] OR "age"[Title/Abstract] OR "gender"[Title/Abstract] OR "male*"[Title/Abstract] OR "female*"[Title/Abstract] OR "BMI"[Title/Abstract] OR "weight"[Title/Abstract] OR "psychological"[Title/Abstract] OR "social"[Title/Abstract] OR "psychological"[Text Word] OR "social"[Text Word] OR "age"[Text Word] OR "gender"[Text Word] OR "male*"[Text Word] OR "female*"[Text Word] OR "BMI"[Text Word] OR "weight"[Text Word])

Scopus search strategy

## ( TITLE-ABS-KEY ( "Prolonged standing protocol"  OR  "prolonged standing exposure"  OR  "standing exposure"  OR  "LBP inducing protocol"  OR  "Active hip abduction"  OR  "Long term standing"  OR  "long-term standing"  OR  "Prolonged standing"  OR  "Sustained standing"  OR  "Continuous standing"  OR  "standing-induced pain paradigm"  OR  " 2 hour standing"  OR  "2 h standing"  OR  "2 hour standing protocol"  OR  "prolonged standing test"  OR  "2 hours of standing"  OR  "standing for 2 hours"  OR  "2 h trial*"  OR  "2 h"  OR  "2 hours" ) )  AND  ( TITLE-ABS-KEY ( "Risk factor*"  OR  "Contributing factor*"  OR  "predisposing factor*"  OR  posture  OR  "Muscle activation"  OR  co-activation  OR  "Muscle activity"  OR  "recruitment pattern*"  OR  "Motor control"  OR  instability  OR  "Postural control"  OR  alignment  OR  malalignment  OR  fatigue  OR  kinematics  OR  "Neuromuscular control"  OR  asymmetry  OR  coordination  OR  "Lumbar posture"  OR  "Spinopelvic posture"  OR  "lumbosacral posture"  OR  lordosis  OR  hyperlordosis  OR  hypolordosis  OR  age  OR  gender  OR  male*  OR  female*  OR  bmi  OR  weight  OR  psychological  OR  social ) )  AND  ( ( TITLE-ABS-KEY ( "Pain developer*"  OR  "low back Pain developer*"  OR  "LBP developer*"  OR  "standing intolerant*"  OR  "discomfort developer*"  OR  "LBDiscomfort developer*"  OR  "LBD developer*"  OR  "low back discomfort developer*"  OR  "Prone to LBP"  OR  "susceptible to LBP"  OR  "disposed to LBP"  OR  "Prone to low back pain"  OR  "susceptible to low back pain"  OR  "disposed to low back pain"  OR  lbpd*  OR  "develop* LBP"  OR  "develop* low back pain" ) )  OR  ( TITLE-ABS-KEY ( "Low back pain"  OR  "LBP"  OR  "back pain"  OR  "low back discomfort"  OR  "Transient low back pain"  OR  "transient LBP"  OR  "standing induced low back pain"  OR  "standing induced LBP"  OR  "standing induced pain"  OR  "Induced pain"  OR  "Induced pain paradigm"  OR  "induced low back pain paradigm"  OR  "induced LBP paradigm"  OR  "induced LBP"  OR  "inducing LBP"  OR  "inducing low back pain"  OR  "Perceived pain"  OR  "Subjective discomfort" ) ) )

WOS search strategy

AB=("Pain developer*"  OR  "low back Pain developer*"  OR  "LBP developer*"  OR  "standing intolerant*"  OR  "discomfort developer*" OR “LBDiscomfort developer*” OR “LBD developer*” OR “low back discomfort developer*” OR "Prone to LBP"  OR  "susceptible to LBP"  OR  "disposed to LBP"  OR  "Prone to low back pain"  OR  "susceptible to low back pain"  OR  "disposed to low back pain"  OR  LBPD*  OR  "develop* LBP"  OR  "develop* low back pain") OR AB=("Low back pain"  OR  "LBP"  OR  "back pain"  OR “low back discomfort” OR "Transient low back pain"  OR  "transient LBP"  OR  "standing induced low back pain"  OR  "standing induced LBP"  OR  "standing induced pain"  OR  "Induced pain"  OR  "Induced pain paradigm"  OR  "induced low back pain paradigm"  OR  "induced LBP paradigm"  OR  "induced LBP"  OR  "inducing LBP"  OR  "inducing low back pain"  OR  "Perceived pain"  OR  "Subjective discomfort") AND AB=("Prolonged standing protocol"  OR “prolonged standing exposure” OR “standing exposure” OR “LBP inducing protocol” OR "Active hip abduction"  OR  "Long term standing"  OR  "long-term standing"  OR  "Prolonged standing"  OR  "Sustained standing"  OR  "Continuous standing"  OR  "standing-induced pain paradigm"  OR  " 2 hour standing"  OR  "2 h standing"  OR "2 hour standing protocol" OR "prolonged standing test"  OR  "2 hours of standing"  OR  "standing for 2 hours"  OR  "2 h trial*" OR "2 h" OR "2 hours") AND AB=("Risk factor*"  OR  "Contributing factor*" OR “predisposing factor*" OR  posture  OR  "Muscle activation"  OR  co-activation  OR  "Muscle activity"  OR  "recruitment pattern*"  OR  "Motor control"  OR  instability  OR  "Postural control"  OR  alignment  OR  malalignment  OR  fatigue  OR  kinematics  OR  "Neuromuscular control"  OR  asymmetry  OR  coordination  OR  "Lumbar posture"  OR  "Spinopelvic posture"  OR  "lumbosacral posture"  OR  lordosis  OR  hyperlordosis  OR  hypolordosis  OR  age  OR  gender  OR  male*  OR  female*  OR  bmi  OR  weight  OR  psychological  OR  social)

**Supplementary Material 2**

Table 1. Checklist for assessing the quality of quantitative studies

| Criteria | | Yes  (2) | Partial  (1) | No  (0) | NA |
| --- | --- | --- | --- | --- | --- |
| 1 | Question / objective sufficiently described? |  |  |  |  |
| 2 | Study design evident and appropriate? |  |  |  |  |
| 3 | Method of subject/comparison group selection or source of information/input variables described and appropriate? |  |  |  |  |
| 4 | Subject (and comparison group, if applicable) characteristics sufficiently described? |  |  |  |  |
| 5 | If interventional and random allocation was possible, was it described? |  |  |  |  |
| 6 | If interventional and blinding of investigators was possible, was it reported? |  |  |  |  |
| 7 | If interventional and blinding of subjects was possible, was it reported? |  |  |  |  |
| 8 | Outcome and (if applicable) exposure measure(s) well defined and robust to measurement / misclassification bias? Means of assessment reported? |  |  |  |  |
| 9 | Sample size appropriate? |  |  |  |  |
| 10 | Analytic methods described/justified and appropriate? |  |  |  |  |
| 11 | Some estimate of variance is reported for the main results? |  |  |  |  |
| 12 | Controlled for confounding? |  |  |  |  |
| 13 | Results reported in sufficient detail? |  |  |  |  |
| 14 | Conclusions supported by the results? |  |  |  |  |
|  | Summary score |  |  |  |  |

Note, N/A is not a response option for items for items 1, 2, 4, 10 and 11. The summary score was calculated as: total sum[(number of ‘yes’ × 2) + (number of ‘partial’ × 1)]/total possible sum[22 − (number of ‘N/A’ × 2)], with a maximum possible total score of 1

**Supplementary material 3**  Compilation of studies and their corresponding proportion of individuals that PDs and NPDs

| Code | Author(s) | Population | Total sample | | | Gender of PDs | | Gender of NPDs | | Percentage of PDs (%) | Note (I) | Duration of standing (II) |
| --- | --- | --- | --- | --- | --- | --- | --- | --- | --- | --- | --- | --- |
|  |  |  | Total (III) | PDs | NPDs | Male | Female | Male | Female |  | Code | 2 h |
| 1 | Nelson-Wong et al. (2008) [1] | University | 23 | 15 | 8 | N/A | N/A | N/A | N/A | 65.21% |  | 2 h |
| 2 | Gregory et al. (2008) [2] | University | 13 | 7 | 6 | 4 | 3 | 2 | 4 | 53.8% |  | 2 h |
| 3 | Nelson-Wong et al. (2009) [3] | University and surrounding community | 43 | 17 | 26 | 7 | 10 | 15 | 11 | 39.53% |  | 2 h |
| 4 | Nelson-Wong and Callaghan (2010) [4] (IV) | University and surrounding community | 43 | 17 | 26 | 7 | 10 | 15 | 11 | 39.53% | 3 | - |
| 5 | Nelson-Wong and Callaghan (2010) [5] | University and surrounding community | 43 | 20 | 23 | N/A | N/A | N/A | N/A | 46.5 | 3 | - |
| 6 | Nelson-Wong et al. (2010) [6] | University and surrounding community | 43 | 17 | 26 | 7 | 10 | 15 | 11 | 39.53% | 3 | - |
| 7 | Nelson-Wong and Callaghan (2010) [7] | University and surrounding community | 23 | 8 | 15 | 3 | 5 | 8 | 6 | 34.78% | 3 | - |
| 8 | Nelson-Wong and Callaghan (2010) [8] | University and surrounding community | 16 | 8 | 8 | 4 | 4 | 4 | 4 | 50% | 3 | - |
| 9 | Callaghan et al. (2010) [9] (V) | University | 16 | 6 | 10 | N/A | N/A | N/A | N/A | 37.5% | 3 | - |
| 10 | Gallagher et al. (2011) [10] | University and surrounding community | 41 | 13 | 27 | 4 | 9 | 16 | 11 | 31.7% |  | 2 h |
| 11 | Marshall et al. (2011) [11] | University | 24 | 17 | 7 | N/A | N/A | N/A | N/A | 71% |  | 2 h |
| 12 | Nelson-Wong et al. (2012) [12] | University and surrounding community | 43 | 17 | 26 | N/A | N/A | N/A | N/A | 39.53% | 3 | - |
| 13 | Raftry and Marshall (2012) [13] | University | 20 | 10 | 10 | N/A | N/A | N/A | N/A | 50% |  | 2 h |
| 14 | Gallagher et al. (2014) [14] | N/A | 20 | 11 | 9 | N/A | N/A | N/A | N/A | 55% |  | 45 min (sat for 15 min and repeated this sequence) |
| 15 | Gallagher (2014) [15] |  | 23 | 12 | 11 | 7 | 5 | 4 | 7 | 52.1% | 19,23 | - |
| 16 | Marshall et al. (2014) [16] | N/A | 26 | 16 | 10 | N/A | N/A | N/A | N/A | 61.5% |  | 2 h |
| 17 | Sorensen et al. (2015) [17] | Universities and surrounding community | 57 | 24 | 33 | 9 | 15 | 20 | 13 | 42.1% |  | 2 h |
| 18 | Aghazadeh et al. (2015) [18] | University | 15 | 10 | 5 | 10 | N/A | 5 | N/A | 66.6% |  | 2 h |
| 19 | Gallagher and Callaghan (2015) [19] | University | 32 | 14 | 18 | 10 | 4 | 7 | 11 | 43.75% |  | 2 h |
| 20 | Sorensen et al. (2016) [20] | Universities and surrounding community | 57 | 24 | 33 | 9 | 15 | 20 | 13 | 42.1% | 17 |  |
| 21 | Sorensen et al. (2016) [21] | Universities and surrounding community | 57 | 24 | 33 | 9 | 15 | 20 | 13 | 42.1% | 17 |  |
| 22 | Gallagher et al. (2016) [22] | University | 17 | 9 | 11 | N/A | N/A | N/A | N/A | 52.9% | 19 |  |
| 23 | Gallagher and Callaghan (2016) [23] | N/A | 17 | 9 | 11 | 4 | 5 | 5 | 3 | 52.9% | 75 min |  |
| 24 | Viggiani and Callaghan (2016) [24] | University | 40 | 16 | 24 | 8 | 8 | 12 | 12 | 40% | 20 |  |
| 25 | Stewart and Gregory (2016) [25] | University | 16 | 12 | 4 | N/A | N/A | N/A | N/A | 75% | 2 h |  |
| 26 | Viggiani et al. (2017) [26] |  | 16 | 8 | 8 | 5 | 3 | 4 | 4 | 50% | 19 |  |
| 27 | Fewster et al. (2017) [27] | N/A | 23 | 11 | 12 | N/A | N/A | N/A | N/A | 47.8% | 2 h |  |
| 28 | Naseri and Kahrizi (2017) [28] | University students | 25 | 10 | 15 | 7 | 3 | 6 | 9 | 40% | 2 h |  |
| 29 | Hwang (2018) [29] | University and local community | 71 | 33 | 38 | 16 | 17 | 19 | 19 | 46% | 2 h |  |
| 30 | Hwang et al. (2018) [30] | University and local community | 71 | 33 | 38 | 16 | 17 | 19 | 19 | 46% | 28 |  |
| 31 | Lee et al. (2018) [31] | Convenience sample through personal and professional networks | 18 | 14 | 4 | N/A | N/A | N/A | N/A | 77.7% | 2 h |  |
| 32 | Lurie et al. (2018) [32] | N/A | 18 | 13 | 5 | N/A | N/A | N/A | N/A | 72.2% | 2.5 h |  |
| 33 | Viggiani and Callaghan (2018) [33] | University | 40 | 16 | 24 | 8 | 8 | 12 | 12 | 40% | 24 |  |
| 34 | Misir et al. (2018) [34] | N/A | 38 | 20 | 18 | N/A | N/A | N/A | N/A | 52.6% | 2 h |  |
| 35 | Cardenas and Gregory (2018) [35] | University | 15 | 7 | 8 | N/A | N/A | N/A | N/A | 46.6% | 2 h |  |
| 36 | Park (2018) [36] | Universities and surrounding community | 39 | 17 | 22 | 9 | 8 | 10 | 12 | 43.5% | 2 h |  |
| 37 | Wall et al. (2019) [37] (VI) | N/A | 55 | 22 | 33 | N/A | N/A | N/A | N/A | 40% | 4.5 h |  |
| 38 | Fewster et al. (2019) [38] | N/A | 12 | 6 | 6 | N/A | N/A | N/A | N/A | 50% | Not said |  |
| 39 | Edgar et al. (2019) [39] | Universities and surrounding community | 31 | 14 | 17 | N/A | N/A | N/A | N/A | 45.25% | 2 h |  |
| 40 | Weber (2019) [40] | N/A | 40 | 20 | 20 | 8 | 12 | 11 | 9 | 50% | 105 min |  |
| 41 | Gallagher et al. (2019) [41] | N/A | 19 | 11 | 8 |  |  |  |  | 57.8% | 2 h |  |
| 42 | Viggiani et al. (2020) [42] (VII) | N/A | 21 | 10 | 11 | 4 | 6 | 5 | 6 | 47.6% | 2 h |  |
| 43 | Viggiani et al. (2020) [43] | N/A | 17 | 8 | 9 | 5 | 4 | 3 | 5 | 47% | 2 h |  |
| 44 | Nelson-Wong et al. (2020) [44] | Employees in Denver/Metro region | 32 | 20 | 12 | 12 | 8 | 4 | 8 | 62.5% | 2 h |  |
| 45 | Fewster et al. (2020) [45] | University | 32 | 13 | 19 | N/A | N/A | N/A | N/A | 42% | 2 h |  |
| 46 | Orakifar et al. (2021) [46] | University | 32 | 13 | 19 | 5 | 8 | 9 | 10 | 40.62% | 1 h |  |
| 47 | Khoshroo et al. (2021) [47] | University | 60 | 30 | 30 | N/A | 30 | N/A | 30 | 50% | 2 h |  |
| 48 | Johnston et al. (2021) [48] | University | 20 | 16 | 4 | N/A | 16 | N/A | 4 | 80% |  |  |
| 49 | McKinnon et al. (2021) [49] | University | 24 | 12 | 12 | 8 | 4 | 5 | 7 | 50% | 2 h |  |
| 55 | Homaei-Morad (2022) [50] | University | 33 | 15 | 18 | N/A | 15 | N/A | 18 | 45.45% | 2 h |  |
| 51 | Alghosi (2022) [51] | University | 33 | 16 | 17 | 16 | N/A | 17 | N/A | 48.48% | 2 h |  |
| 52 | Winberg et al. (2022) [52] | University students | 16 | 8 | 8 | 4 | 4 | 4 | 4 | 50% | 1 h |  |
|  | Total (VIII) |  | 1070 | 528 | 542 | 142 (IX) | 181 | 163 | 194 | 49% |  |  |
|  | Abbreviation: h , hour; min, minute | | | | | | | | | | | |

1. Some studies have utilized same participants from a previous investigation or they are different reports of same participants’ dataset. This part indicates references to the related studies.
2. It refers to the duration of time when participants performed prolonged standing in order to be classified as PDs and NPDs.
3. It refers to the number of participants whose data have been analyzed.
4. Discrepancies in the number of PDs presented within the journal articles exist; the number of PDs were originally 20 but after recalculating VAS with removal of baseline, 3 PDs were categorized into non-PDs (Nelson-Wong, 2009, p. 178-179).
5. The sample was 24. 16 out of 24 participants performed prolonged standing protocol.
6. Nine of the 55 included subjects reported low back complaints in the previous 12 months and an additional four had lower back complaints during the past seven days, none of whom felt impeded in their regular jobs.
7. The sample was 32. 11 participants incurred recurrent LBP and 21 participants were either PDs or NPDs.
8. It excludes the studies using same participants from previous investigation or they are different reports of same participants’ dataset.
9. Studies that did not report sex were excluded.

**Supplementary material 4**

PDs and NPDs’ common and distinctive characteristics pre-, during, and post-standing

|  | Outcome | Phase | Significant differences between PDs and NPDs | Non-significant differences between PDs and NPDs |
| --- | --- | --- | --- | --- |
| Muscle |  |  |  |  |
|  | Stiffness | Pre |  | - No difference in hamstring extensibility, passive stiffness, and stretch tolerance [13] |
|  |  | Post |  | - No difference in hamstring extensibility, passive stiffness, and stretch tolerance [13] |
|  | Strength | Pre | -Less MVC and more between two sides asymmetry at the left plantar flexors in PDs [28] | -A trend towards lower strength in baseline strength values of standing maximal isometric standing hip abduction contractions in PDs (p = .092) [33], but no difference in lying hip abduction strength measures [11, 36]  - No difference in MVC of dorsi flexors, abductors and adductors and extensors and flexors of the hip, flexors and abductors of the core, flexors and abductors and extensors of the shoulder [28] |
|  |  | Post |  | -Significant decrease in side-lying hip abduction strength for both groups [11] |
|  | Endurance | Pre | - *Lower endurance of side-lying, repetitive (dominant) leg raising exercise with similar strength losses [33] and side-bridge in PDs [11] | - No difference in GMed muscle endurance during the reverse side-bridge [36] and side support [3] |
|  |  | Post | -Lower side-bridge endurance time in PDs [11] |  |
|  | Fatigue | pre |  | -No difference in baseline Median frequency (MF) measurements for right third lumbar erector spinae (L3ES) [31]  -No difference in mean power frequency (MPF) decreases with fatigue after the side-lying, repetitive (dominant) leg raising exercise for bilateral GMed and tensor facia lata (TFL) [33] |
|  |  | During |  | -No significant change over the time or between the two groups in MF of right L3ES [31]  -Almost no differences in lumbar muscle activity (except for the 5th percentile of surface electromyography, which was higher in NPDs). (11.7%RVE vs. 7.5%RVE) [37] |
|  |  | Post | -PDs’ inability to recover force losses after 120 minutes of standing after the side-lying, repetitive (dominant) leg raising exercise performed before prolonged standing [33]  -A greater rate of fatigue for contralateral GMed during the side-bridge test in PDs [11] |  |
|  | oxygenation |  |  | -No difference in near infrared spectroscopy measures on the right L3ES with no significant change over time, and poor correlations with the standing-induced perceived low back discomfort scores (r = 0.07) [9] |
|  | Muscle activity | Pre | -A greater average number of responsive extensor muscles and a greater occurrence of extensor muscle response due to trunk perturbations with a 6.78 kg load suddenly applied to the hands [2] |  |
|  |  | Post | -A greater average number of responsive extensor muscles and a greater occurrence of extensor muscle response due to trunk perturbations with a 6.78 kg load suddenly applied to the hands [2] |  |
|  | GMed co-activation | Pre | -Higher GMed co-activation in PDs [27] |  |
|  |  | During | - Consistent higher co-activation of the bilateral GMed muscles in PDs [1, 11, 18]  -Higher levels of muscle co-activation in PDs only during the first (before subjective report of pain) and final 30 min of 2-h standing period [4]  -Strong negative correlation between VAS score and co-activation index for the bilateral GMed during the acute pain development (30–90 min) (r = -0.73) [4] |  |
|  | Trunk muscle co-activation | Pre |  | -No significant differences in cross-correlation values for right and left LES [27] |
|  |  | During | -Higher LLES–LEO co-activation in PDs [1]  -Higher levels of trunk muscle co-activation in PDs at the beginning and end of the 2-h standing period [4]  -An increase of trunk muscle co-activation in NPDs while a decrease in PDs during the acute pain development (30–90 min) of 2-h standing period with a strong negative correlation with VAS score ( r = -0.92) [4]  - More day-to-day variability in trunk muscle coactivation in NPDs [7] | -Neither difference nor change over time in co-activation patterns of the thoracic erector spinae with rectus abdominus or external obliques, bilateral thoracic erector spinae, lumbar erector spinae with rectus abdominus, or bilateral lumbar erector spinae muscles [1] |
|  | Muscle coordination | Pre |  | -No significant difference in anticipatory and compensatory postural adjustments of deep abdominal muscles during rapid shoulder flexion and extension and no association with standing-induced LBP [16] |
|  |  | Post | Standing-induced LBP was associated with the changes in APA amplitudes post-standing (rs = 0.43, p = 0.002) [16] | -Reductions in the anticipatory muscle activity of deep abdominals for both groups during shoulder flexion [16]. |
| Posture |  | | | |
|  | Lumbar posture | Pre | -*larger lumbar lordosis in PDs [17, 34] and a significant relationship with Max VAS (r = 0.46, P = 0.02) [17]  -A more even distribution of intervertebral angles in upright standing poses throughout PDs’ lumbar spines and proportionately less lumbar lordosis at lower levels of the lumbar spine and proportionately more lordosis at higher lumbar levels [26] | -No significant difference in lumbar lordosis [22, 26, 29] and lumbar angle (lumbar spine with respect to pelvis) [23]  - No significant relationship between baseline horizontal distances between the spinous processes of the T6 and L3 to the LBP measures [29]  -No significant difference in lumbosacral lordosis angle, L1/L2 intervertebral joint angle, L5/S1 intervertebral joint angle [22, 34] though NPD had 3° more extension with their LSL angles (p = 0.1882) [22]  - No significant difference in the ranges of motion at each lumbar intervertebral level and similar relative positioning of upright standing between the end-range poses and vertebral shape, and recurve (related to lumbar spine flexion) and no relation of them to pain development in standing [26] |
|  |  | During | -Significant correlation of the horizontal distance measures between the spinous processes of the T6 and L3 over time to LBP intensity in PDs (r value range: 0.31-0.42, p value range: 0.01-0.04) [29]  -*The association of larger lumbar lordosis within PDs with a higher Max VAS pain [40]  - Less movement of PDs’ lumbar spine and increased thoracic extension in contrast to greater thoracic flexion (by approximately 8 deg) in NPDs [14]  -Less range of lumbar spine angle in PDs [14]  -Lower frequency of lumbar spine flexion/extension fidgets in PDs [19]  -Less lumbar spine fidgets in the sagittal plane in PDs (p ¼ 0.0412) [19, 23] | - No significant differences in standing posture lumbar lordosis [29, 40]  -Identical lumbar spine angle with respect to their maximum lumbar spine extension angle [14]  -No significant relationship between lumbar curvature to LBP over time during standing [29]  - No significant difference in UL and LL end-range range posture with slightly decreased UL lordosis over time away from usual standing posture in both groups [31]  -No significant differences and changes for both UL and LL postural movement [31]  - No significant differences in lumbar spine movements (shifts and fidgets) between PDs and NPDs [52] |
|  | Lumbar spine passive stiffness | Pre | -Standing in the state of higher lumbar spine passive stiffness with a lumbar spine angle further beyond their passive lumbar spine neutral zone compared to NPDs [15] |  |
|  | Trunk | Pre |  | -No significant difference in trunk angle (trunk with respect to pelvis) (However, PDs stood 4 degrees further away from the maximum trunk extension angle than NPDs (p = 0.1037) [23].) |
|  | Hip and thigh | Pre |  | -No significant difference in right /left hip angle (thigh with respect to pelvis), right/left trunk-thigh angle (thigh with respect to trunk) (during 60-sec constrained standing) [23] |
|  | pelvic posture | Pre | -Larger pelvic incidences and sacral slopes in PDs [34] | -No differences in pelvic tilt [34] |
|  |  | During |  | -No difference in LP angle [52] |
|  | COP | Pre | -Greater PDs’ mean COP displacement during 60-s quiet standing [46] | -No significant difference in averaged and maximum COPAP location over 50 ms prior to the perturbation with a 6.78 kg load suddenly applied to the hands [2]  - No significant differences in anterior-posterior postural sway (in both static and dynamic modes), medial-lateral postural sway (in both static and dynamic positions) and general postural sway [50, 51] |
|  |  | During | -Greater ML fidget frequency in PDs [10]  - PDs’ further location of COP to the heel during the first 15 min [10]  -PDs’ lower COPAP and COPML range, and Velocity AP and Velocity ML range [32] | -No significant difference in COP A/P fidget and shift frequency [19]  - No significant difference in COP variables that tended to increase, albeit minimally, over time such as range in the CoPAP , range in CoPML, CoPAP_RMS, CoPML_RMS, and CPL [35]  -No significant differences in CoP variables between the end-point PDs and NPDs [32] |
|  | Linear COP measures | Post | -NPDs’ greater increase in AP MPF during the eyes open 2-min constrained standing task [45]  -An increase in PDs’ MPF while no change in NPDs’ AP MPF during the eyes closed 2-min constrained standing task [45]  -A significant increase in COP ML MPF during the eyes opened 2-min constrained standing task for both groups, but a greater change in NPDs [45]  -Increased COP displacement and velocity in ML direction in PDs during 60-s standing on the foam surface compared to NPDs and compared to standing on the firm surface [46]  -Increased COP displacement in ML direction during 60-s standing on the foam surface compared to before the prolonged standing [46]  -Greater mean COP displacement in ML direction during quiet standing on the firm condition than standing on the foam condition in NPDs [46]  -Lower mean velocity during standing on the firm condition in PDs compared to before the prolonged standing (P = 0.051) [46] | A significant increase in COP AP MPF during the eyes opened 2-min constrained standing task for both groups [45] |
|  | nonlinear COP measure | Post | -A greater COP regularity (decreased sample entropy) in PDs [45] |  |
|  | BWS | During | -Less large body weight transfers (30% BW) during the first 15 min in PDs [19]  -PDs increased their large weight-shift frequency more consistently compared with NPDs [44] | -An increase in the BW shift frequency over the 2-h protocol in both groups [10, 19, 44] and only in male participants [24], and a decrease in average shift duration over the 2-h protocol in both groups [10] |
|  | Proprioceptive postural control strategies | Pre | -PDs’ less reliance on lumbar multifidus proprioception than that of triceps surae during 60 s quiet standing (altered proprioceptive postural control strategies) [46] |  |
|  |  | Post |  | - No significant difference in proprioceptive postural control strategies [46] |
| Movement Patterns/pre | | | | |
| AHAbd | | Pre | -Earlier movement of lumbopelvic region earlier during left hip abduction than right hip abduction in PDs and significant correlation with average symptom intensity during standing (r = 0.46; p = 0.02) [21]  -Lower movement smoothness in PDs exhibiting larger angular displacement arc length during AHA performance (worse performance) with an external weight at the ankle relative to no external weight [36]  -*Higher (worse) examiner-rated AHAbd scores in PDs [3, 47, 50, 51] having 3.85 (95% confidence interval [CI]: 1.05-19.07) [3] or even 18.3 (CI 95%, 3.674–91.22) [47] times higher odds of developing LBP during the PSP with a positive test  -Self-rated AHAbd as more difficult by PDs having 6.55 (95% CI: 1.14- 37.75) times higher odds of developing LBP during the PSP with a positive [3] | -No difference in Angular Velocity Arc Length [36]  -No difference in Active Hip abduction ROM [21]  -No difference in examiner-rated AHAbd scores [36] |
| Symmetric Floor-To-Knuckle Lifting | |  |  | -No significant difference in spine motion, frontal plane knee motion, and muscle sequencing patterns [36] |
| Modified Star Excursion Balance Test Reach Distances | |  |  | -No significant difference in dynamic balance control and frontal plane knee excursions [36] |
| Standing lumbar extension task (lean, static and return) | |  | -More co-activity in PDs’ hip extensors (GMax, long head of Biceps Femoris) [42] | -No significant difference in ranges of motion during trunk extension, movement control strategies, and movement coordination [42] |
| Standing maximum lumbar extension | |  | -More even distribution of intervertebral angles in full extension throughout lumbar spines with the lack of lower segment contributions in PDs spread throughout the upper lumbar levels (L1-L4) compared to NPDs with larger contributions from lower lumbar segments (L5-S1) [26]  -Larger (more extended) lumbar lordosis, L1/L2 IV angle, and sacral slope in PDs [34] | -No difference in LSL, L5/S1 IV angle, and pelvic tilt [34] |
| FMS | |  | - lower (worse) scores in the composite and individual component FMS score in PDs [47]  -Discrimination of PDs and NPDs with the optimal cutoff scores of ≤ 14 on the FMS, 2 on the push-up, and 1 on the deep squat [47]  - Association of lower FMS scores with an earlier onset of and higher LBP intensity during standing [47]  -PDs with at least one bilateral asymmetry on the FMS had 10 times (95% CI, 2.941–34.008) and with at least two bilateral asymmetries on the FMS had 15.5 times (95% CI, 3.814–63.359) higher odds of developing LBP during prolonged standing than NPDs [47]  -Negative correlation of LBP intensity with LBP onset (rs (30) = –0.509, p = 0.004) during the PSP [47] |  |
| Maximal trunk flexion-extension exertions | |  | Faster performance maximal trunk flexion-extension exertions by NPDs [36] |  |
| Submaximal lumbar flexion-extension | | Pre | -Heightened relaxation response of the hip extensors (gluteus maximus muscles) during forward flexion at the trunk in PDs [6]  - *A ‘top-down’ muscle recruitment strategy with lumbar extensors activated prior to gluteus maximus in PDs for return-to-stand from forward flexion [12]  -Elevated erector spinae-external oblique (RES-LEO) coactivation during return-to-stand in PDs [39]  -Greater GMax and LES muscle activity in PDs during flexion phase (lower flexion relaxation) [25]  -Elevated erector spinae-internal oblique coactivation with Sacro-iliac joint (SIJ) bracing during return-to stand in PDs [39] | -No significant difference in typical muscle recruitment strategy (bottom-up) [36, 39], lumbopelvic kinematics [36], relative trunk (thorax relative to pelvis), global pelvis and global thorax angles [6], and trunk angular velocity magnitudes and lumbar/hip ratios [12, 36] for return-to-stand from forward flexion  -No differences in relaxation response of the lumbar extensors during forward flexion [6]  - Reversed extensor sequencing with SIJ bracing during return-to-stand in both groups [39] |
|  | | Post | -Heightened relaxation response for the gluteus maximus muscles in PDs [6] | -No significant difference in relative trunk (thorax relative to pelvis), global pelvis and global thorax angles [6] |
| Unloaded squat | | Pre |  | -No difference in relative trunk (thorax relative to pelvis), global pelvis and global thorax angles [6] |
|  | | Post |  | - No significant difference in relative trunk (thorax relative to pelvis), global pelvis and global thorax angles [6]  -No significant differences in the peak or average total ‘active’ vertebral joint rotation stiffness values in either the ‘down’ or ‘up’ phases of the squat [6]  -A slight decrease in lumbar erector spinae’s relative contribution to ‘active’ rotational stiffness about the flexion/extension axis [6]  -No significant findings on the individual muscle (LES, TES, LAT, RA, IO, EO, GMed, GMax) contributions to ‘active’ stiffness during either the ‘down’ or ‘up’ phases of squatting [6] |
| Single-leg stance | | Pre | -Higher hip abductor activity in non-dominant unilateral stance, and increased hip extensor activity with SIJ bracing in NPDs [39] | -No significant difference in relative trunk (thorax relative to pelvis), global pelvis and global thorax angles, and center of pressure excursion [6] |
|  | | Post | -Lower peak activation of left GMed in NPDs and higher in PDs during left single-leg standing [6]  -Increased trunk extension in PDs in contrast to increased flexion in NPDs [6]  -Increased global pelvis lateral bend in PDs and decreased global pelvis lateral bend in NPDs during left single-leg standing [6] | -Lower peak activation in females and higher peak activation levels in males during right single-leg standing [6]  -Increased relative trunk angle in lateral bending in both groups [6]  -No difference in COP excursion [6]  -Increased COP excursion during single leg standing in males [6]  -Decreased active stiffness in lateral bend at L2/L3, and L4/L5 in 10-second right single-leg standing for both groups [6]  -No significant differences in peak ‘active’ rotational stiffness during either right or left single-leg standing [6]  -No significant differences in peak or average individual muscle (LES, TES, LAT, RA, IO, EO, GMed, GMax) contributions to rotational stiffness [6] |
| Stair decent | |  | - larger lumbar lordosis in PDs [34] | -No significant differences in LSL, L1/L2 and L5/S1 IV angles, pelvic tilt, and sacral slope [34] |
| Stair climbing | |  | - Larger lumbar lordosis, and sacral slope in PDs [34] | -No differences in pelvic tilt, L1/L2, L5/S1, LSL [34] |
| Sitting | | During | -No large difference in lumbar spine flexion range of motion between sitting and standing in PDs [14]  -Similar median thoracic angles with the angle increasing by approximately 10 of greater flexion in sitting in PDs compared to standing in contrast to the NPDs’ consistent posture even when entering into sitting [14]  -More extended lumbar spines and L1/L2 intervertebral (IV) angle in PDs [34] | -No differences in LSL, L5/S1 IV angle, pelvic tilt, and sacral slope [34]  -Identical lumbar spine angle with respect to their maximum lumbar spine extension angle [14] |
|  | | Leaning forward while sitting | - More prominent flexion of LL, LSL and L5/S1 IV angles compared to upright standing posture in PDs [34] | -No significant differences in lumbar lordosis, L1/L2 and L5/S1 IV angles, pelvic tilt, and sacral slopes [34] |
| Gait /walking | | During |  | -Increased lumbar region range of motion in the coronal and transverse planes during walking in both groups [41]  -Increased median lumbar flexion during walking in both groups [41]  -No significant difference in stance phase duration of gait [50, 51] |
| Joint | | | | |
| Ankle | | Pre |  | -Similar ankle self-reported function [36] |
| Psychological variables | | | | |
|  | | Pre | -*Higher (worse) scores in Pain Catastrophizing Scale (PCS) in PDs [36]  *-Higher (worse) scores in Fear of Pain Questionnaire (FPQ-III) in PDs [36] and a greater Fear of Pain minor subscale scores predicted greater initial LBP and a more rapid increase in LBP over the 2 hours of standing [29]  -*Large and significant correlation between average standing-induced LBP intensity and FPQ-III (r=0.91, p=.03), and large for PCS but non-significant (r=0.87, p=.06) for only PDs with a maximum VAS score ≥20 mm (clinically important level of LBP) [20] | -No significant difference in PCS [20, 28, 30] and SOPA-B (Survey of Pain Attitudes - Brief) [4]  -No significant difference in FPQ-III [20] and FABQ (Fear Avoidance Beliefs Questionnaire) [4]  -Insignificant correlation between FPQ-III and PCS scores and LBP intensity experienced [29]  -No significant difference in CRPP (Cognitive Risk Profile for Pain) [4], Hospital Anxiety (HADS Anxiety)  Depression Scale (HADS Depression) [30], anxiety inventory (STAI) and Tampa Scale for kinesiophobia (TSK) [28] |
| Structural variables | | | | |
| Intervertebral disks | | Supine and pre | - Smaller A/P ratios of each lumbar IVD (less wedging) in female PDs and male NPDs than female NPDs and male PDs, respectively [40] | -No significant difference in the degenerative status of IVDs between PDs and NPDs at any of the lumbar levels [40] |
| Anthropometric variables | | | | |
| Trunk to height ratio | | Pre | -A greater trunk to height ratio predicted a greater and faster increase in LBP intensities over the 2 hours of standing in PDs [29] |  |
| BMI | |  | -Even with the recruiting criteria of BMI < 30, the larger the BMI, the greater the severity of the pain symptoms [40] |  |
| Age, gender, and standing habituation | |  |  | -No moderating effect of age, gender, or standing habituation on development of LBD during standing [37] |
| Breast size | |  | -Higher amounts of pain and erector spinae co-activation at the upper, middle, and low back in female PDs with large breast size [48] |  |
| Sensory variables | | | | |
| Sensory processing | | Pre |  | -No significant differences in sensory processing using Quantitative sensory testing (QST) (thermal thresholds, wind-up ratio, mechanical pain pressure threshold, and conditioned pain modulation) [30] |

**Supplementary material 5**

PDs and NPDs’ distinctive characteristics by gender

|  | Variable | Phase | Significant differences between male and female PDs/NPDs | Significant differences between males and females |
| --- | --- | --- | --- | --- |
| Muscle |  |  |  |  |
|  | Endurance | Pre |  | - Longer endurance times in females with smaller strength losses immediately following the fatiguing exercise [33] |
|  | Strength | Pre |  | -Higher baseline strengths in males [33] |
| Posture |  |  |  |  |
|  |  | Pre | - Greater neutral zone length in male PDs than male NPDs and vice versa for females [15] | -Two times higher moment to the location of their neutral zone boundary for both flexion and extension in males than females’ corresponding moment [15] |
|  | COP shift, fidget, drift | During | - A lower average of ML drift frequency and drift amplitude in male PDs than the other three groups [10]  -Much smaller average AP shift amplitude in male PDs during the first 15 min of standing, followed by a large variation for the remainder of the protocol in contrast to the other groups showing consistency over the 2 h [10] | -Greater AP drift frequency in females than males [10] |
|  | BWS | During | -An even number of shifts between their right and left leg in female PDs in contrast to female NPDs and male PDs’ more shifts onto their left leg [10]  - Male PDs spent the least amount of time in an asymmetrical posture. Male PD spent less than 10% of the first 15 min in an asymmetrical stance, followed by a gradual increase in asymmetry during the first hour and then became consistent with male NPD for the second hour (20–30%) [10] | -Increased small and large BWTs with time for all males not for females [24] |
|  | Lumbar fidgets | During |  | - More lumbopelvic fidgets in females than males during each 15-min interval [52] |
| Movement patterns | during a seated chair-tilting task while cyclically tilting their pelvises anteriorly and posteriorly on an unstable chair |  | -Larger normalized lordosis range in male PDs in the anterior direction compared with male NPDs and female PDs [43]  -Higher lordosis ranges and peak lordosis extension angular velocities in female NPDs than male NPDs [43]  - Higher ranges in female NPDs than male NPDs and female PDs when tilting anteriorly [43]  - A greater range of intervertebral angles in female NPDs when tilting anteriorly compared with tilting posteriorly [43]  - Greater L5/S1 contributions to extension in female PDs than female NPDs [43]  -Higher peak velocities in female NPDs than male NPDs and female PDs when tilting anteriorly [43]  -Faster peak extension velocities in female NPDs when tilting anteriorly compared with tilting posteriorly [43] |  |
| Structural |  |  | -Smaller A/P ratios of each lumbar IVD (less wedging) in female PDs and male NPDs than female NPDs and male PDs, respectively [40] | -Larger IVD widths in males than females [40] |
| Intervention |  |  |  |  |
|  | Self-selected alternation between standing on a 16-degree incline and decline surface | during |  | -Decreased global trunk flexor/extensor CCI during platform standing in females [8] |
|  | During 124- min protocol of standing work with seated breaks at a 3:1 stand-sit ratio with increasing durations from 3:1 minute to 48:16 minutes. | during |  | -Greater left GMD activation and consistently greater CCI in females than males [49] |
|  | A hip abductor fatigue exercise before prolonged standing | During | -Earlier significant reductions in LBP (after 90 min) for females despite reporting more LBP than males ( after 120 min) [24]  -More anterior pelvic tilt and greater lumbar spine extension in male PDs and more posterior pelvic tilt and greater lumbar spine flexion in female PDs during the fatigue session compared to the control session [24] |  |
|  | 60-min of standing while doing light manual work on an AFM | During |  | -More lumbopelvic fidgets in females during each 15-min interval [40] |

**Supplementary material 6**

The effect of intervention studies on distinctive variables between PDs and NPDs pre-, during, and post-standing

| Intervention | Assessment | Variable | Findings of standing intervention |
| --- | --- | --- | --- |
| Foot rest | | | |
| 5-min standing on an elevated surface [27] | Changes in lumbar spine posture and muscular activity using motion capture system and EMG, respectively | Muscle    Posture | There were no significant differences in Gmed co-activation (cross-correlation values) between PDs and NPDs as a result of an increase in NPDs’ Gmed co-activation even slightly higher than PDs’. They also had similar right and left LES co-activation.  All participants stood in a more flexed lumbar spine posture compared to level ground. |
| An elevated surface [22] | The influence of an elevated foot rest on lumbopelvic posture using sagittal radiography | Posture | Lumbosacral lordosis and L5/S1 intervertebral joint flexion occurred when elevating one leg onto a raised platform; however, there were no significant differences in lumbopelvic measures between PDs and NPDs. |
| 5-min standing on an elevated surface [15] | Median lumbar spine angle for level and elevated standing using a motion capture system | Posture | The elevated surface brought all participants’ lumbar spine into flexion and closer to the passive neutral zone, and thus reduced the associated lumbar spine passive stiffness in both pain developers and non-pain developers. |
| 80-min 3-1-3-1 protocol cycling through 3-min level ground standing and one-min leg raise [38] | Lumbar spine joint angles and GMed cross-correlation values using a motion capture system and EMG, respectively | Pain  Muscle  Posture | Only one out of the six previously known PDs was still classified as a PD.  There were no significant differences in Gmed co-activation (cross-correlation values) between PDs and NPDs. It also provided a break in the co-activation profiles of the GM exhibited by PDs during level ground standing. Both displayed a trend of significantly higher GM cross-correlation values when one leg was elevated in comparison to level standing.  It Increased lumbar spine flexion in comparison to level standing during both the intervals of elevated leg standing and level standing over time. |
| 2-h protocol having the right foot and then the left foot raised on the footrest, and then both on the floor each for 5 min [31] | Muscle fatigue of the right erector spinae using EMG and low back end-range posture and movement using a motion capturing system compared to 2-h level standing control session | Pain  Posture | It did not significantly decrease LBD, however LBD reported with and without a footrest appeared to be diverging at 2 h (51.1 mm (±24.2 mm) without a footrest and 32.0 mm (±15.2 mm) with the footrest). LBD seemed to increase more rapidly in the final 30 min in the no footrest condition.  UL posture moved away from usual standing posture without a footrest as it became slightly less lordotic. Whilst UL lordosis became more similar to usual standing over time with footrest use as it became more lordotic. There were no changes in muscle fatigue over time and postural movements. |
| Anti-fatigue mat (AFM) | | | |
| 2-h of standing on an AFM [18] | GMed co-activation using sEMG compared with 2-h standing on rigid floor | Pain  Muscle | Although AFM significantly decreased subjective pain level, it failed to significantly decrease the number of PDs.  It slightly increased NPD’s GMed co-activation and decreased PDs’. However, it objectively did not significantly change the GMed co-activation pattern related to LBP. |
| 60-min of standing on an AFM [52] | Lumbo-pelvic angles, lumbar shifts and fidgets, and foot-floor interface such as COP excursion, COP A/P fidgets, COP A/P Shifts, BW Shifts using a motion capture system and two force platforms, respectively compared with 1-h standing on rigid floor | Pain  posture | The amount of standing-induced LBP was significantly reduced in the PDs when on the AFM (3.6 ± 6 mm) compared to the rigid floor (6.8 ± 7 mm).  The AFM increased total COP movement, with both PDs and NPDs exhibiting greater COP excursions on the AFM compared to the rigid floor condition (NPD 55% increase; PD 35% increase). AFM tended to increase the number of BWS by 116% in NPDs and 54% in PDs. There was a trend of increase in M/L body weight shifts involving greater than 50% of a weight transfer (p = 0.062). However, there was a trend to induce less movements in ML weight shifts ( 10–30% BW) in PDs (p = 0.07) [52]. |
| Vibrating belt at the low back | | | |
| 3-min vibration every 12 minutes during 2-h standing [35] | COP variables using a force plate compared with 2-h standing without vibrating belt on the control day | Pain  posture | LBP prior to each vibration bout was significantly higher than that immediately following, suggesting a temporary relief of pain in PDs. However, the level of perceived LBP at the end of the 2 h on the control day was not significantly different from that on the vibration day.  Only NPDs showed the drop in cumulative path length on the experimental day. CoP variables such as COP AP, range in COP ML, COP AP_RMS, COP ML_RMS, and CPL tended to increase, albeit minimally, over time. The range of the COP ML velocity was not affected by time and velocity ML tended to vary substantially over time. COP ML_RMS and CPL tended to be highest on the control day and lowest on the experimental day when the vibration was on. Decreases in anterior-posterior and medial-lateral center of pressure movement were also observed during each bout of vibration compared to those during the control day. |
| 3-min vibration applied at the 2-h and 2.25-h marks during a 2.5-h standing task [32] | COP variables 3 min at the start of the standing period and then for four additional 3-min periods starting at time (in minutes) 105 (no vibration), 120 (during first bout of vibration), 135 (during second bout of vibration), and 147 min (no vibration). | Pain  posture | PDs reported statistically lower VAS scores compared to prior to the vibration following each vibration bout, however, LBP returned to pre-vibration levels when the vibration ceased.  In general, NPDs had greater COP AP and COP ML range and the Velocity AP and Velocity ML than mid-point PDs; however, the drop in these variables as a result of vibration was similar between the pain groups. There were no significant differences in COP variables between end-point PDs and NPDs. |
| Fatigue protocol | | | |
| side-lying, repetitive (dominant) leg raising exercise before prolonged standing [24] | COP measures of each foot using force plates calculating large and small body weight transfer compared to control (without exercise beforehand) session | Pain  Posture  Muscle | PDs reported lower peak VAS scores during the fatigue session. Female reductions began after 90 min and male reductions began after 120 min.  Male PDs exhibited more anterior pelvic tilt and lumbar extension during the fatigue session compared to the control session and female PDs had more posterior pelvic tilt and lumbar flexion than female NPDs during the fatigue session. Also, fatigue increased movement responses in all participants with larger APMIs (calculated by the shift and fidget frequencies) during the 30-min block of the fatigue session compared to the control session.  Both the small and large BWTs increased with time during the 30-min block for all participants with a similar trend extending to the 45-min block  PDs’ R-GMD/ L-GMD CCIs reduced and NPDs’ increased with fatigue during the first 15 min of standing. However, differences were not present ever after. |
| side-lying, repetitive (dominant) leg raising exercise before prolonged standing [33] | Hip abductor strength, fatigability, and recovery during prolonged standing after fatigue compared to control (without exercise beforehand) session | Muscle | PDs’ hip abductors fatigued faster than NPDs with similar perceived effort and force losses and mean power frequency (MPF) decreased similarly with fatigue for bilateral GMed and TFL before 2-h standing. Unlike NPDs, PDs did not recover force losses after 120 minutes of standing. Myoelectric changes with fatigue reverted to unfatigued levels after ten minutes of standing and were not useful in differentiating PDs and NPDs. |
| Declined surface | | | |
| 5-min standing on a declining sloped surface of 16 degrees [27] | Changes in lumbar spine posture and muscular activity using a motion capture system | Muscle  Posture | There were no significant differences in Gmed co-activation (cross-correlation values) between PDs and NPDs. This was as a result of an increase in cross-correlation values for NPDs. They also had similar right and left LES co-activation.  There were no significant kinematic differences (lumbar angles) between declined sloped condition and level ground. |
| Sloped surface of 16 degrees [22] | Lumbopelvic posture using sagittal radiography | Posture | There was no difference in lumbar spine angle when standing on level ground versus a decline. Declined sloped surface induced L1/L2 intervertebral joint flexion. |
| 5-min standing on a declining sloped surface of 16 degrees [15] | Self-selected lumbar spine angle compared to the location of the lumbar spine passive neutral zone between standing on a declining sloped surface and level ground using a motion capture system | Posture | The sloped surface did not affect the relationship between the lumbar spine angle and neutral zone location. |
| 60-sec trial and 75-min prolonged standing on declining sloped surface of 16 degrees [23] | Acute and over-time postural changes compared to 60-sec and 75-min level-ground standing | Pain  Posture | PDs’ average maximum pain scores were 58% lower during sloped standing.  60-sec standing trial on the sloped surface significantly induced hip flexion, trunk-to-thigh flexion and moved the location of the trunk center of gravity posteriorly (p < 0.0001) by 1.9 cm.  Lumbar spine fidgets were unchanged when standing on the sloped surface with NPDs performing significantly more lumbar spine fidgets in the sagittal plane than PDs. The trunk center of gravity moved posteriorly and vertically aligned with the right ankle joint for all participants compared to level ground standing. |
| Self-selected alternation between standing on a 16 degrees incline and decline surface [8] | Muscular and biomechanical responses using EMG and motion analysis system | Pain  Muscle  Posture | Using a sloped surface reduced LBP scores by 59.4% for PD when compared to level standing.  It decreased PDs’ gluteus medius co-activation levels, but increased NPDs’.  The sloped surfaces also created a favorable minimal postural variability in both pelvic and lumbar spine angles. The incline surface introduced anterior rotation of the pelvis and a corresponding increase in lumbar spine extension and knee extension. The decline surface resulted in the opposite postural shift with posterior rotation of the pelvis and an increase in flexion of the lumbar spine and knee flexion. |
| Staggered standing | | | |
| The arch of the front foot was in line with the toes of the other foot for 5 min [27] | Changes in lumbar spine posture and muscular activity using a motion capture system and EMG | Muscle  Posture | There were no significant differences in Gmed co-activation (cross-correlation values) between PDs and NPDs. This was as a result of an increase in cross-correlation values for NPDs. They also had similar right and left LES co-activation.  There were no significant kinematic differences (lumbar angles) between staggered sloped condition and level ground. |
| Staggered standing for 5 min [15] | Self-selected lumbar spine angle compared to the location of the lumbar spine passive neutral zone between standing on a declining sloped surface and level ground using a motion capture system | Posture | staggered stance did not affect the relationship between the lumbar spine angle and neutral zone location. |
| Forward bending while standing | | | |
| Bending forward to full spine flexion for 5 s every 15 min at the start and during 2-h standing [25] | Lumbar spine posture, erector spinae and GMed muscle activation using motion capture system and surface EMG for comparison with the control 2-h standing session | Pain  Muscle | Intermittent trunk flexion decreased LBP by 36% (10 mm) at the end of a 2-h standing and at 75 min onward, post-flexion LBP scores became significantly lower than control day flexion scores. After the full 120 min, pre-flexion LBP scores became significantly lower than control day scores.  Lumbar Erector spinae and GMed muscle relaxation during FR was reported in 91% and 65% of the flexion trials respectively, indicating that periods of rest occurred possibly contributing to the reduction in LBP observed. |
| Sitting | | | |
| 12-week graded standing exposure using a sit-stand desk and home exercise for the intervention group and sit-stand desk without instruction or home exercise for the control group [44] | Standing small and large body weight shifts compared between the intervention and control groups during 2-h standing prior to and following the intervention | Pain  Posture | All PDs in intervention and control groups had decreased LBP regardless of intervention. (Day 1 average VAS = 10.43 ± 1.65 mm, Day 2 average VAS = 2.3 ± 4.0 mm, Day 1 maximum VAS = 21.9 ± 3.73 mm, Day 2 maximum VAS = 5.1 ± 6.9 mm)  All PDs adopted a more dynamic strategy with significantly increased large shift frequency after 12-weeks while NPDs had no significant change. |
| A 3:1 stand to sit ratio during two 1-hour blocks (45-min standing followed by a 15-min sitting) [14] | Thoracic and lumbar spine angles using three tri-axial accelerometers placed on T1, L2 and S2 to compare between PDs and NPDs during standing and sitting | Pain  Posture | When PDs transitioned to a seated position, they demonstrated an average decrease of 12.5 mm (7 mm) in their subjective LBP reports and upon standing again demonstrated an increase of 16.4 mm (9.2 mm) over the 45 minutes to a higher level than was present in the first 45 minutes of standing. A 15-minute seated break failed to provide lasting recovery of LBP from standing.  PDs moved through a limited range of their lumbar spine angle and increased thoracic extension resulting in static postures. There was not a significant difference in the ROM of lumbar spine flexion between sitting and standing in PDs. |
| 124-min of standing work with seated breaks at a 3:1 stand-sit ratio with increasing durations from 3:1 minute to 48:16 minutes [49] | Muscular activation, co-activation, and spine posture measures during the protocol between previously classified PDs and NPDs | Pain  Posture  Muscle | The protocol could successfully reduce LBPs in PDs to the level of NPDs’, with mean pain scores of 13 mm which was only slightly higher than the clinical LBP threshold of 10 mm.  Spine posture measures showed no differences between PDs and non-PDs.  PDs demonstrated slightly but significantly lower activation in their left LES muscle than NPDs. There were greater left GMD activation and consistently greater coactivation in females. |
| 4.5 h over three periods with two seated breaks (110-min standing followed by 35-min sitting, 110-min standing followed by 10-min sitting, and 55-min standing) [37] | Lumbar muscle activity and pelvic movement using surface EMG and a gravimetrical position sensor compared between PDs and NPDs | Pain  Muscle  Posture | Levels of discomfort intensity increased up to a median of two at the end of each of the three standing exposures, and reduced after the breaks back to a median of zero.  There were almost no differences in lumbar muscle activity except for the 5th percentile of surface EMG that was higher in NPDs.  NPDs demonstrated a statistically significant increase of medio-lateral pelvic movement in the progression of the three standing exposures. |
| Exercise | | | |
| 4-week progressive stabilization-based exercise program [5] | Trunk and GMed muscle activation responses to a prescribed exercise using EMG compared between the intervention and control pain groups during 2-h standing prior to and following the intervention. | Pain  Muscle | PDs in the intervention group had significantly lower VAS scores (8.93 ± 3.66 mm) than PDs of the control group (16.5 ± 6.3 mm).  Only male PDs in the intervention group had significantly decreased GMed co-activation levels and increased gap length of the right GMed muscle following 4-week exercise intervention. |
| Walking | | | |
| 5-minute walking breaks every 25 min during 2-h standing [41] | Lumbar region kinematics angle and range of motion using motion capture system compared  with the control 2-h standing session | Pain  Posture | 70% of PDs were categorized as NPDs during 2-h standing with walking breaks (22.1 (10.8) mm compared to 6.4 (1.8) mm).  There were no kinematic differences between PDs and NPDs. Median lumbar flexion and lumbar region range of motion in the coronal and transverse planes increased during walking compared to standing for both groups. |

**Supplementary material 7**: Forest plots


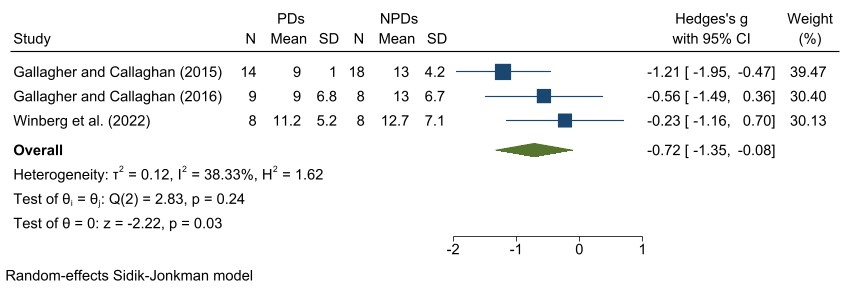


Figure A: Forest plot of comparison: association of lumbar fidgets with standing-induced LBP


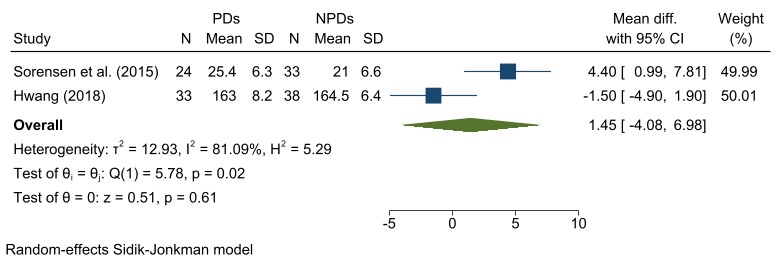


Figure B: Forest plot of comparison: association of LCA with standing-induced LBP


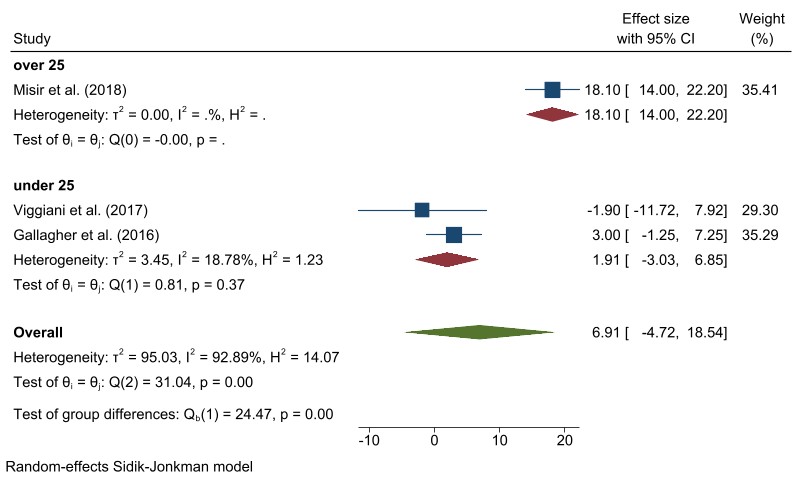


Figure C: Forest plot of comparison: association of lumbar Cobb angle with standing-induced LBP


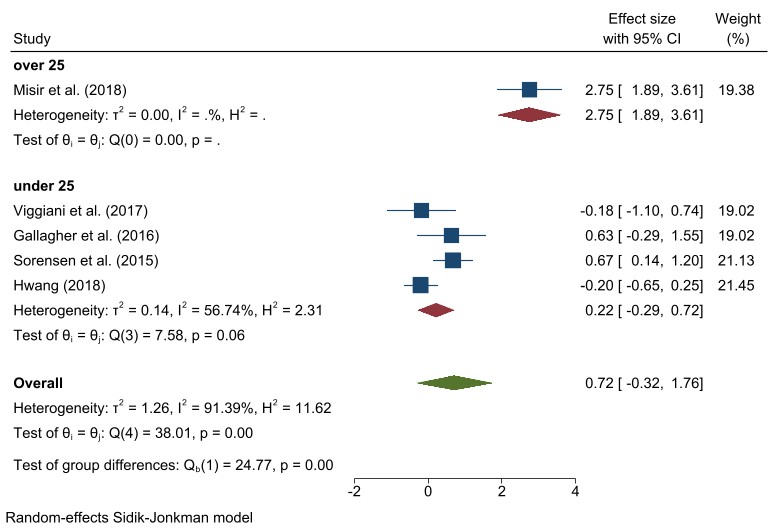


Figure D: Forest plot of comparison: association of lumbar lordosis with standing-induced LBP


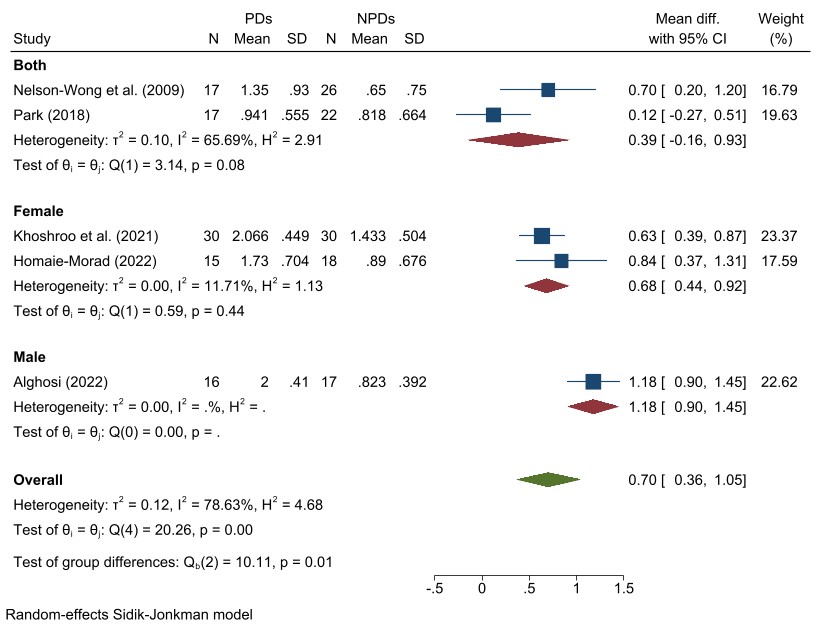


Figure E: Forest plot of comparison: association of AHAbd test with standing-induced LBP


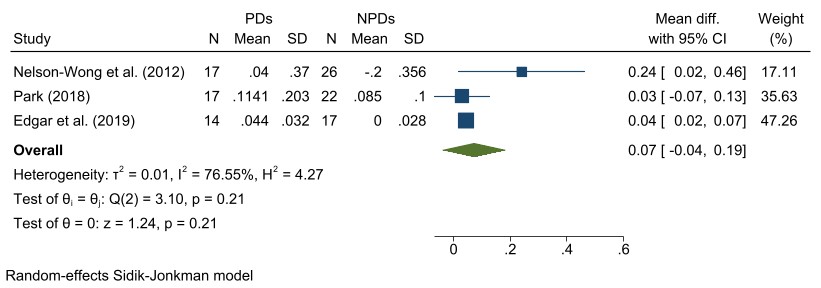


Figure F: Forest plot of comparison: association of muscle recruitment strategy for return-to-stand from forward flexion with standing-induced LBP


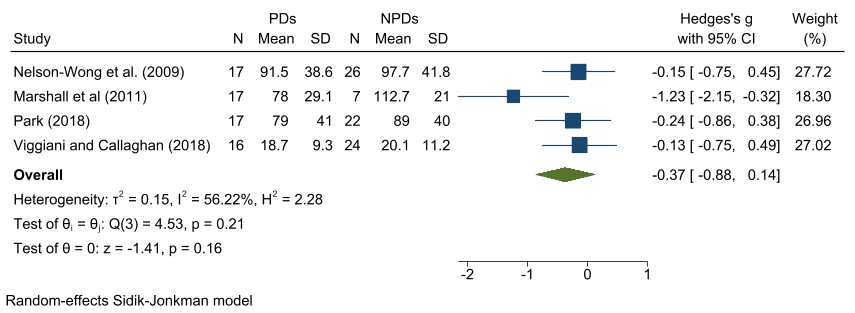


Figure G: Forest plot of comparison: association of hip abductor endurance with standing-induced LBP


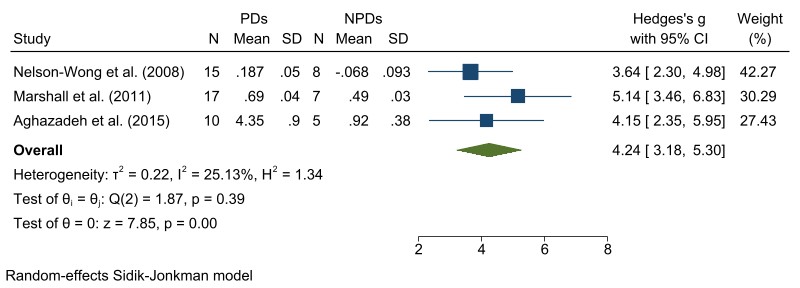


Figure H: Forest plot of comparison: association of GMed co-activation with standing-induced LBP


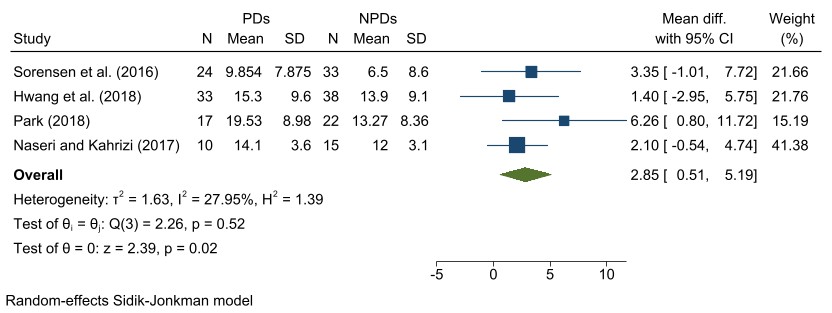


Figure I: Forest plot of comparison: association of PCS with standing-induced LBP


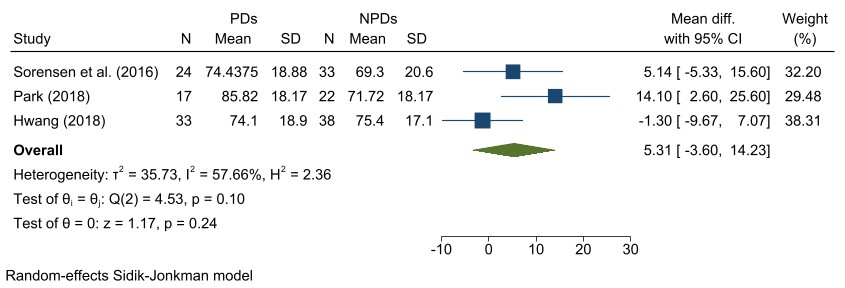


Figure J: Forest plot of comparison: association of FPQ-III with standing-induced LBP

1. Nelson-Wong, E., et al., *Gluteus medius muscle activation patterns as a predictor of low back pain during standing.* Clin Biomech (Bristol, Avon), 2008. **23**(5): p. 545-53.

2. Gregory, D.E., S.H. Brown, and J.P. Callaghan, *Trunk muscle responses to suddenly applied loads: do individuals who develop discomfort during prolonged standing respond differently?* J Electromyogr Kinesiol, 2008. **18**(3): p. 495-502.

3. Nelson-Wong, E., T. Flynn, and J.P. Callaghan, *Development of active hip abduction screening test for identifying occupational low back pain.* Journal of Orthopaedic and Sports Physical Therapy, 2009. **39**(9): p. 649-657.

4. Nelson-Wong, E. and J.P. Callaghan, *Is muscle co-activation a predisposing factor for low back pain development during standing? A multifactorial approach for early identification of at-risk individuals.* J Electromyogr Kinesiol, 2010. **20**(2): p. 256-63.

5. Nelson-Wong, E. and J.P. Callaghan, *Changes in muscle activation patterns and subjective low back pain ratings during prolonged standing in response to an exercise intervention.* J Electromyogr Kinesiol, 2010. **20**(6): p. 1125-33.

6. Nelson-Wong, E., S.J. Howarth, and J.P. Callaghan, *Acute biomechanical responses to a prolonged standing exposure in a simulated occupational setting.* Ergonomics, 2010. **53**(9): p. 1117-28.

7. Nelson-Wong, E. and J.P. Callaghan, *Repeatability of clinical, biomechanical, and motor control profiles in people with and without standing-induced low back pain.* Rehabilitation research and practice, 2010. **2010**.

8. Nelson-Wong, E. and J.P. Callaghan, *The impact of a sloped surface on low back pain during prolonged standing work: a biomechanical analysis.* Appl Ergon, 2010. **41**(6): p. 787-95.

9. Callaghan, J.P., D.E. Gregory, and J.L. Durkin, *Do NIRS measures relate to subjective low back discomfort during sedentary tasks?* International Journal of Industrial Ergonomics, 2010. **40**(2): p. 165-170.

10. Gallagher, K.M., E. Nelson-Wong, and J.P. Callaghan, *Do individuals who develop transient low back pain exhibit different postural changes than non-pain developers during prolonged standing?* Gait Posture, 2011. **34**(4): p. 490-5.

11. Marshall, P.W., H. Patel, and J.P. Callaghan, *Gluteus medius strength, endurance, and co-activation in the development of low back pain during prolonged standing.* Hum Mov Sci, 2011. **30**(1): p. 63-73.

12. Nelson-Wong, E., et al., *Altered muscle recruitment during extension from trunk flexion in low back pain developers.* Clin Biomech (Bristol, Avon), 2012. **27**(10): p. 994-8.

13. Raftry, S.M. and P.W. Marshall, *Does a 'tight' hamstring predict low back pain reporting during prolonged standing?* J Electromyogr Kinesiol, 2012. **22**(3): p. 407-11.

14. Gallagher, K.M., T. Campbell, and J.P. Callaghan, *The influence of a seated break on prolonged standing induced low back pain development.* Ergonomics, 2014. **57**(4): p. 555-62.

15. Gallagher, K.M., *The relationships of prolonged standing induced low back pain development with lumbopelvic posture and movement patterns.* 2014.

16. Marshall, P.W., R. Romero, and C. Brooks, *Pain reported during prolonged standing is associated with reduced anticipatory postural adjustments of the deep abdominals.* Exp Brain Res, 2014. **232**(11): p. 3515-24.

17. Sorensen, C.J., et al., *Is lumbar lordosis related to low back pain development during prolonged standing?* Man Ther, 2015. **20**(4): p. 553-7.

18. Aghazadeh, J., et al., *Anti-fatigue mats, low back pain, and electromyography: An interventional study.* Int J Occup Med Environ Health, 2015. **28**(2): p. 347-56.

19. Gallagher, K.M. and J.P. Callaghan, *Early static standing is associated with prolonged standing induced low back pain.* Hum Mov Sci, 2015. **44**: p. 111-21.

20. Sorensen, C.J., et al., *Psychological Factors Are Related to Pain Intensity in Back-Healthy People Who Develop Clinically Relevant Pain During Prolonged Standing: A Preliminary Study.* Pm r, 2016. **8**(11): p. 1031-1038.

21. Sorensen, C.J., et al., *Asymmetry of lumbopelvic movement patterns during active hip abduction is a risk factor for low back pain development during standing.* Hum Mov Sci, 2016. **50**: p. 38-46.

22. Gallagher, K.M., M. Sehl, and J.P. Callaghan, *A radiographic assessment of lumbar spine posture in four different upright standing positions.* Clin Biomech (Bristol, Avon), 2016. **37**: p. 131-136.

23. Gallagher, K.M. and J.P. Callaghan, *Standing on a declining surface reduces transient prolonged standing induced low development.* Applied Ergonomics, 2016. **56**: p. 76-83.

24. Viggiani, D. and J.P. Callaghan, *A hip abduction exercise prior to prolonged standing increased movement while reducing cocontraction and low back pain perception in those initially reporting low back pain.* J Electromyogr Kinesiol, 2016. **31**: p. 63-71.

25. Stewart, D.M. and D.E. Gregory, *The use of intermittent trunk flexion to alleviate low back pain during prolonged standing.* J Electromyogr Kinesiol, 2016. **27**: p. 46-51.

26. Viggiani, D., et al., *The distribution of lumbar intervertebral angles in upright standing and extension is related to low back pain developed during standing.* Clin Biomech (Bristol, Avon), 2017. **49**: p. 85-90.

27. Fewster, K.M., K.M. Gallagher, and J.P. Callaghan, *The effect of standing interventions on acute low-back postures and muscle activation patterns.* Appl Ergon, 2017. **58**: p. 281-286.

28. Naseri, S. and S. Kahrizi, *Plantar flexor muscles asymmetry and their lower strength is maybe related to development of low back pain during prolonged standing.* Journal of Clinical Physiotherapy Research, 2017. **2**(3): p. 133-138.

29. Hwang, C.-T., *Biological and Psychological Factors Contributing to the Development and Course of Low Back Pain in Prolonged Standing.* 2018.

30. Hwang, C.T., L.R. Van Dillen, and S. Haroutounian, *Do Changes in Sensory Processing Precede Low Back Pain Development in Healthy Individuals?* Clin J Pain, 2018. **34**(6): p. 525-531.

31. Lee, J.Y., et al., *Use of a footrest to reduce low back discomfort development due to prolonged standing.* Appl Ergon, 2018. **67**: p. 218-224.

32. Lurie, R.C., et al., *The effect of short duration low back vibration on pain developed during prolonged standing.* Appl Ergon, 2018. **67**: p. 246-251.

33. Viggiani, D. and J.P. Callaghan, *Hip Abductor Fatigability and Recovery Are Related to the Development of Low Back Pain During Prolonged Standing.* J Appl Biomech, 2018. **34**(1): p. 39-46.

34. Misir, A., et al., *Lumbar spine posture and spinopelvic parameters change in various standing and sitting postures.* European Spine Journal, 2019. **28**(5): p. 1072-1081.

35. Cardenas, A. and D.E. Gregory, *Can cutaneous vibration affect pain development? Testing the efficacy of a vibrating belt applied intermittently to the low back region during prolonged standing.* International Journal of Industrial Ergonomics, 2018. **66**: p. 95-IBC.

36. Park, J., *Assessing pre-existing movement and muscular recruitment differences in prolonged standing, transient low back pain developers compared to non-pain developers*. 2018, University of Waterloo.

37. Wall, R., et al., *Associations between low back muscle activity, pelvic movement and low back discomfort development during prolonged standing - An exploratory laboratory study.* International Journal of Industrial Ergonomics, 2019. **72**: p. 380-389.

38. Fewster, K.M., et al., *Does proactive cyclic usage of a footrest prevent the development of standing induced low back pain?* Hum Mov Sci, 2019. **66**: p. 84-90.

39. Edgar, K., et al., *Influence of Sacroiliac Bracing on Muscle Activation Strategies During 2 Functional Tasks in Standing-Tolerant and Standing-Intolerant Individuals.* J Appl Biomech, 2019. **35**(2): p. 107-115.

40. Weber, C.I., *﻿ Structural and Tissue-level Adaptations of Lumbar Intervertebral Discs in Humans with Inducible Low Back Pain Symptoms*. 2019: Washington University in St. Louis.

41. Gallagher, K.M., et al., *Walking breaks can reduce prolonged standing induced low back pain.* Hum Mov Sci, 2019. **66**: p. 31-37.

42. Viggiani, D., et al., *A comparison of trunk control in people with no history, standing-induced, and recurrent low back pain during trunk extension.* Journal of Manual & Manipulative Therapy, 2020. **28**(2): p. 94-102.

43. Viggiani, D., et al., *Lumbar Intervertebral Kinematics During an Unstable Sitting Task and Its Association With Standing-Induced Low Back Pain.* J Appl Biomech, 2020: p. 1-13.

44. Nelson-Wong, E., et al., *Increasing standing tolerance in office workers with standing-induced back pain.* Ergonomics, 2020. **63**(7): p. 804-817.

45. Fewster, K.M., et al., *Low back pain development differentially influences centre of pressure regularity following prolonged standing.* Gait Posture, 2020. **78**: p. e1-e6.

46. Orakifar, N., et al., *Comparison of proprioceptive postural control strategies between prolonged standing induced low back pain developers and non-low back pain developers.* Physiotherapy Theory and Practice, 2021.

47. Khoshroo, F., et al., *A comparison of functional movement patterns between female low back pain developers and non-pain developers.* Work, 2021. **69**(4): p. 1247-1254.

48. Johnston, H., S. Wanninayake, and J.D. Drake, *Investigating women’s chest size, trunk muscle co-contraction and back pain during prolonged standing.* Journal of Back and Musculoskeletal Rehabilitation, 2021. **34**(3): p. 371-380.

49. McKinnon, C.D., D.R. Martel, and J.P. Callaghan, *The impact of a progressive sit-stand rotation exposure duration on low back posture, muscle activation, and pain development.* Ergonomics, 2021. **64**(4): p. 502-511.

50. Homaei-Morad, N., *Comparison of postural sway and duration of the stance phase of gait between female low back pain developers and non-pain developers*, in *Health and Sports Medicine Department*. 2022, University of Tehran.

51. Alghosi, M., *Comparison of postural sway and duration of the stance phase of gait between male low back pain developers and non-pain developers*, in *Health and Sports Medicine Department*. 2022, University of Tehran.

52. Winberg, T.B., et al., *Anti-fatigue mats can reduce low back discomfort in transient pain developers.* Appl Ergon, 2022. **100**: p. 103661.
